# Design and Construction of Portable CRISPR-Cpf1-Mediated Genome Editing in *Bacillus subtilis* 168 Oriented Toward Multiple Utilities

**DOI:** 10.3389/fbioe.2020.524676

**Published:** 2020-09-02

**Authors:** Wenliang Hao, Feiya Suo, Qiao Lin, Qiaoqing Chen, Li Zhou, Zhongmei Liu, Wenjing Cui, Zhemin Zhou

**Affiliations:** ^1^The Key Laboratory of Industrial Biotechnology, Ministry of Education, School of Biotechnology, Jiangnan University, Wuxi, China; ^2^Jiangnan University (Rugao) Food Biotechnology Research Institute, Jiangsu, China

**Keywords:** CRISPR-Cpf1, *Bacillus subtilis*, multiplex genome editing, large fragment deletion, gene insertion, chassis microorganisms

## Abstract

*Bacillus subtilis* is an important Gram-positive bacterium for industrial biotechnology, which has been widely used to produce diverse high-value added chemicals and industrially and pharmaceutically relevant proteins. Robust and versatile toolkits for genome editing in *B. subtilis* are highly demanding to design higher version chassis. Although the *Streptococcus pyogenes* (*Sp*) CRISPR-Cas9 has been extensively adapted for genome engineering of multiple bacteria, it has many defects, such as higher molecular weight which leads to higher carrier load, low deletion efficiency and complexity of sgRNA construction for multiplex genome editing. Here, we designed a CRISPR-Cpf1-based toolkit employing a type V Cas protein, Cpf1 from *Francisella novicida.* Using this platform, we precisely deleted single gene and gene cluster in *B. subtilis* with high editing efficiency, such as *sacA*, *ganA, ligD* & *ligV*, and *bac* operon. Especially, an extremely large gene cluster of 38 kb in *B. subtilis* genome was accurately deleted from the genome without introducing any unexpected mutations. Meanwhile, the synthetic platform was further upgraded to a version for multiplex genome editing, upon which two genes *sacA* and *aprE* were precisely and efficiently deleted using only one plasmid harboring two targeting sequences. In addition, we successfully inserted foreign genes into the genome of the chassis using the CRISPR-Cpf1 platform. Our work highlighted the availability of CRISPR-Cpf1 to gene manipulation in *B. subtilis*, including the flexible deletion of a single gene and multiple genes or a gene cluster, and gene knock-in. The designed genome-editing platform was easily and broadly applicable to other microorganisms. The novel platforms we constructed in this study provide a promising tool for efficient genome editing in diverse bacteria.

## Introduction

*Bacillus subtilis*, a well-characterized Gram-positive bacterium, has been regarded to be a “generally recognized as safe” (GRAS) microbe that can naturally secrete numerous extracellular proteins (Correa et al., 2020). *B. subtilis* is an ideal organism for industrial application, however, the available genetic tools are insufficient compared to other widely used microbial chassis, such as *Escherichia coli* and *Saccharomyces cerevisiae* (Keasling, 2010; Dong and Zhang, 2014). Gene-editing is of great utilization to reprogramming and reshaping the genome of synthetic chassis (Bai et al., 2018). In previous studies, several *B. subtilis* genome editing tools have been developed. Common gene knockout systems in *B. subtilis* include Cre/*loxP* recombination (Suzuki et al., 2005; Choi et al., 2015), MazF counter-selectable markers (Westbrook et al., 2016), and synthetic gene circuits (Jeong et al., 2015). Cre/*loxP* is a recombination system based on resistance selection markers, which can knock out or insert genes by recombining homologous fragments with Cre recombinase. However, this method needs to introduce a foreign resistance gene, which is not in line with the needs of eco-friendly hosts. The counter-selectable method based on MazF knocks out genes by introducing a toxin-antitoxin (TA) system from *E. coli* (Zhang et al., 2006). Although this method does not introduce resistance markers on chromosomes, its efficiency is very low. Recently, gene knockout methods based on synthetic gene circuits have been constructed in *B. subtilis* (Jeong et al., 2015). Although it was unnecessary to introduce foreign resistance marker genes, the efficiency of knocking out large gene clusters is very low. Thus, the genome engineering of *B. subtilis* needs an effective method without antibiotic resistance markers (Suzuki et al., 2005).

Recently, the Class 2 clustered regularly interspaced short palindromic repeat (CRISPR) system has been employed as a powerful tool for genome editing and transcription regulation in many organisms, including bacteria (Jiang et al., 2015; Westbrook et al., 2016; Wu et al., 2018), yeast (Bao et al., 2015), plant (Gao et al., 2017), and mammals (Hwang et al., 2013). CRISPR systems are divided into two categories on the basis of the configuration of their effector molecules (Zetsche et al., 2015). Different from the class 1 CRISPR system, which requires various Cas proteins to coordinate with each other and bind to the crRNA to form a ribonucleoprotein (RNP) complex, class 2 CRISPR system employ a large single-component Cas protein in conjunction with crRNA to mediate genome editing (Zetsche et al., 2015). In type II CRISPR system, Cas9 from *Streptococcus pyogenes* is widely used because it has been studied very clearly. Zhang et al. (2016) constructed the AIO system in *B. subtilis* ATCC 6051a by using Cas9 from *Streptococcus pyogenes.* Using this system, the authors disrupted specific genes (including *srfC*, *spoIIAC*, *nprE*, *aprE*, and *amyE*) in *B. subtilis* ATCC 6051a with 33–53% efficiency (Zhang et al., 2016). The production of β-cyclodextrin glycosyltransferase by modified *B. subtilis* ATCC 6051a (ΔsrfC, ΔspoIIAC, ΔnprE, ΔaprE, and ΔamyE) is more than 2.5 times that of wild-type *B. subtilis* ATCC 6051a. Similarly, Westbrook et al. (2016) developed a CRISPR-Cas9-based toolkit that can knockout, knock-in, knockdown and point mutations of target genes in *B. subtilis*. They employed a strategy of expressing Cas9 and transcribing gRNA on chromosomes, and the authors believe that this method obviates the instability of multicopy plasmid in the host and the pressure of plasmid on the host. CRISPR-Cas9 system requires three essential factors to cleave the genomic DNA: the CRISPR RNA (crRNA), the *trans-*activating CRISPR RNA (tracrRNA), and the Cas9 nuclease (Chen et al., 2017). In this system, crRNA binds partially to the complementary tracrRNA prior to association with Cas9, allowing to form a gRNA-Cas9 complex. The complex identifies specific target site of genomic DNA based on the PAM sequence and generates blunt-ended double strand breakage (DSB) (Hong et al., 2018). Although CRISPR-Cas9 system has achieved huge success in genome editing in different organisms, it has prominent drawbacks, including severe off-target effects and certain unknown toxicity, resulting in low efficiency in a specific case (Fu et al., 2013; Hsu et al., 2013; Jiang et al., 2017; Hong et al., 2018). Recently, an array of novel Cas proteins has been increasingly developed, such as Cpf1 (Zetsche et al., 2015; Hong et al., 2018), Cas12b (Teng et al., 2018; Strecker et al., 2019), and CasX (Liu et al., 2019) from diverse bacteria. Among these Cas proteins, Cpf1 is a protein from bacterial immune system, which has recently been engineered as a genome editing tool in *Clostridium difficile* (Hong et al., 2018), *Corynebacterium glutamicum* (Jiang et al., 2017), and rice (Wang et al., 2017).

Cpf1, derived from a type V CRISPR system, is an effector Cas protein distinct from Cas9 in structure and function (Zetsche et al., 2015). The prominent advantage of Cpf1 over Cas9 is that the maturity of CRISPR arrays does not require additional tracrRNA, so that Cpf1 is able to process pre-crRNA to mature crRNA (Zetsche et al., 2015, 2017). This feature resolves the drawback in the construction of multiple or large expression constructs using Cas9, upon which the procedure of multiplexed-gene editing is possible to be simplified (Zetsche et al., 2015, 2017; Fonfara et al., 2016). Moreover, CRISPR-Cpf1 complexes efficiently cleave the target DNA utilizing a T-rich PAM sequence rather than the G-rich PAM sequence in CRISPR-Cas9 system (Zetsche et al., 2015), which is probably more efficient in *B. subtilis*. In addition, Cpf1 leaves a staggered end with a 5′ overhang after cleaving DNA, which facilitates repairing the nicked DNA by non-homologous end joining (NHEJ) or homology-directed repair (HDR) after cutting (Zetsche et al., 2015; Jiang et al., 2017).

Previous studies have shown that Cpf1 is greatly superior to Cas9 in genome editing (Kim et al., 2016). Compared with Cas9, Cpf1 is a small protein that contains a single well–identified nuclease domain rather than two nuclease domains for Cas9 (Zetsche et al., 2015). For instance, Cpf1 has only 1300 amino acids (Zetsche et al., 2015), which is more suitable to deliver Cas–sgRNA complex. Importantly, Cpf1 is lower in potential toxicity to the host compared to Cas9 (such as *Sp*Cas9) (Jiang et al., 2017). Therefore, it is a more suitable candidate Cas protein for genome editing (Zetsche et al., 2017). In previous studies, CRISPR–Cpf1 system has been engineered as a powerful genome–editing tool and applied to different organisms, including rice (Wang et al., 2017), soybean (Kim et al., 2017), mouse (Hur et al., 2016), zebrafish (Hwang et al., 2013), human cell (Kim et al., 2016), *Mycobacterium smegmatis* (Yan et al., 2017), *yeast* (Buchmuller et al., 2019), and *C. difficile* (Hong et al., 2018). Recently, Wu et al. (2020) constructed a gene editing system based on CRISPR–Cpf1 in *B. subtilis*, and used the system to perform gene knock–out, knock–in, and regulation of target gene expression. The authors employed a two–plasmid model and improved the efficiency of gene editing (including double gene knockout, knock–in, and point mutation) by overexpressing mutated NgAgo^∗^ on plasmids. However, it is unclear whether the efficiency of gene knock-out by CRISPR-Cpf1 system in *B. subtilis* will be varied with the size of the target gene fragment. And it is still to be determined whether CRISPR-Cpf1 system can mediate deletion of large gene cluster in *B. subtilis*.

**FIGURE 1:**
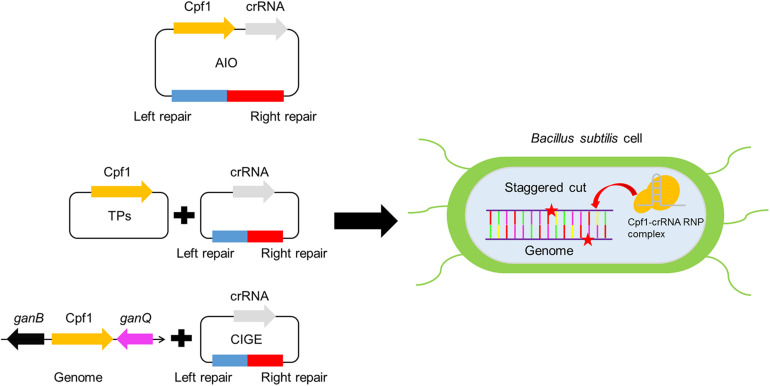
Development of genome editing toolkit based on CRISPR-Cpf1 in *Bacillus subtilis*.

In this study, we broadened genome editing toolkit based on CRISPR-Cpf1 system employing different strategies in *B. subtilis* and successfully applied the CRISPR-Cpf1 system to the deletion of single gene of various sizes as well as multiplex-gene editing in high efficiency (Figure 1). Importantly, we also established an efficient chromosome-integration genome editing (CIGE) platform to achieve precise insertion of gene of interest. These results exhibit that the CRISPR-Cpf1-based tools we have designed and built in this study have highly flexible property that is not only used to deletion of gene of diverse sizes but also serve as a proficient platform to precisely insert heterologous genes into chromosome. This toolbox is of great importance to develop high version of chassis by precisely editing the genome *B. subtilis*, which is great potential to extend the synthetic biology of *B. subtilis*.

## Materials and Methods

### Bacterial Strains and Growth Conditions

All the *E. coli* and *B. subtilis* strains used in this study are listed in Supplementary Table S1. The JM109 clone *E. coli* strains (General Biosystems, China) was used as the general host for plasmid construction and gene cloning. The transformation of the JM109 *E. coli* strains was conducted through chemical transformation using Competent cells from General Biosystems. *E. coli* strains were grown in Luria-Bertani (LB) medium (10 g/L tryptone, 5 g/L yeast extract, 10 g/L NaCl, pH 7.0) supplement with ampicillin (100 μg/mL) or spectinomycin (100 μg/mL) when necessary. Transformation of *B*. *subtilis* cells was carried out by the two-step transformation procedure (Anagnostopoulos and Spizizen, 1961). *B*. *subtilis* strains were cultivated in Luria-Bertani (LB) medium supplement with spectinomycin (100 μg/mL), chloramphenicol (6 μg/mL) or kanamycin (50 μg/mL) and LB solid medium supplemented with 1% glucose.

### Plasmids Construction

All the plasmids used in this study were listed in Supplementary Table S1. All the DNA primers used in this study were listed in Supplementary Table S2. All the crRNA used in this study were listed in Supplementary Table S3. The nucleotide and amino acid sequences of *Fn*Cpf1 and *Sp*Cas9 nucleases were shown in the complementary sequences. The crRNA and sgRNA sequences used in this paper were also listed in the Supplementary Sequences.

### Construction of All-in-One System

The pHTsacA plasmid was derived from pHT01, an expression plasmid for *B. subtilis* (MoBiTec, Göttingen, Germany). P43 promoter and RBS were amplified from pBSG03 using the primers pHT-P43-F and pHT-P43-R while the backbone of the plasmid, harboring *lacI* gene, was amplified using the primer pHT-P43-b-F and pHT-P43-b-R. Then, P43 promoter and RBS were inserted into the shuttle vectors using Gibson assembly (Gibson et al., 2009), yielding pHT-P43-RBS. Accordingly, *Cpf*1 gene was cloned into pHT-P43-RBS using the primers P43-FnCpf1-F/R and P43-FnCpf1-b-F/R, yielding pHT-P43-RBS-FnCpf1. The *sacA*-targeting crRNA expression cassette under the control of a strong promoter P_v__eg_ was synthesized by GENEWIZ Inc., Ltd. (Wuxi, China) and cloned into pHT-P43-RBS-FnCpf1 using the primers pHT-pVeg-sacAcrRNA-F/R and pHT-sacAcr-b-F/R, producing pHT-FnCpf1-*sacA*crRNA. Primer pair sacA-HA-F/R and pHT-HA-b-F/R was used to amplify the 1.2-kb donor DNA template and its bone, respectively. Finally, *sacA* homologous arm was cloned into pHT-FnCpf1-sacAcrRNA, yielding pHTsacA (for the detailed construction method of pHTganA-Cpf1,pHTganA-Cas9, and pHTDV, refer to the Supplementary Method in the Supplementary Material).

The plasmid pHTsfGFPKiT was used to delete *sacA* from chromosome of *B. subtilis*. To construct this plasmid, the sfGFP DNA fragment was amplified from pBPylbp-sfGFP-Ter plasmid using primers sfGFPKi-F/R. The backbone of the plasmid pHTsacA was amplified at the same time using primers pHT-all-sfGFPKi-b-F/R. Then, sfGFP was cloned between the homologous arm of the plasmid pHTsacA, yielding pHTsfGFPKi.

### Construction of Two Plasmids System

To construct gene deletion tool of two-plasmid format, gene *Cpf1* was firstly cloned into pHT01 vector using the primer pHT-Pgrac-Cpf1-F/R and pHT-Pgrac-Cpf1-b-F/R by the Gibson assembly, generating pHT01-Cpf1 activated by Isopropyl-β-D-thiogalactopyranoside (IPTG). To generate the vector for expression of the *sacA*-targeting crRNA, we cloned *sacA*-targeting crRNA expression cassette to pAD123 vector, harboring coding sequences (CDSs) of gfpmut3a and rep60 from pAT1060 origin using the primers pAD123-pVeg-sacAcrT-F/R and pAD123-sacAcrT-b-F/R, generating pAD-pVeg-*sacA*crRNA. The *sacA* homologous arm was cloned into pAD-pVeg-*sacA*crRNA using the primer pAD-sacAH-F/R and pAD-sacAH-b-F/R, yielding pADsacA.

### Construction of Chromosomally Integrated Genome Editing System (CIGE)

To construct a CIGE system that had *Cpf1* integrated in the chromosome in *B. subtilis*, pAX01-Cpf1 under the control of P_xylA_ was constructed to this end using the primer pAX01-Cpf1-F/R and pAX01-Cpf1-b-F/R. Then, rrnBT1 terminator and rrnBT2 terminator was fused downstream of Cpf1 expression cassette. Chloramphenicol-resistant gene (*cat*), *lox*66-71 site and the homologous arm of *ganA* gene was amplified using the primer lacA-Cpf1-F/R, the PCR fragment was integrated specifically into *ganA* site of genome, yielding the strain BS-*ganA’*-Cpf1. To construct plasmid harboring *sacA*-targeting crRNA, we cloned sacAcrRNA expression cassette and *sacA* homologous arm from pHTsacA using the primer pB-pVeg-sacAHA-F/R and pB-pVeg-sacAHA-b-F/R into pBSG03 vector, producing the plasmid pBsacA (for the detailed construction method of pBbac and pBpps, refer to the Supplementary Method in the Supplementary Material).

pBsfGFPKi was constructed to insert super folder green fluorescence protein (sfGFP) into the *sacA* locus. The sacAcrRNA expression cassette, the homologous arms of *sacA*, and the sequence of sfGFP were amplified from pHTsfGFPKi using the primers pB-sacAHA-sfGFP-F/R. Meanwhile, the backbone of the plasmid pHTsfGFPKi was amplified using the primers pB-sacAHA-sfGFP-b-F/R. Accordingly, the two PCR products were fused by Gibson assembly, generating pBsfGFPKi.

The plasmid pB-sacAKo-aprEKi was constructed by one-pot Golden Gate assembly reaction (Engler et al., 2009). Specifically, pB-sacAcrRNA-HA used primers AmpM-*Bsa*I-F/R and RepM-*Bsa*I-F/R to mutate ampicillin-resistance gene and repB replication gene to eliminate *Bsa*I recognition sites. The purpose to do this was to prevent the *Bsa*I restriction enzyme from non-specifically cutting other positions in the reaction of Golden Gate assembly. Then, we amplified the 500-bp homologous arms upstream and downstream of *aprE* from the plasmid pB-aprEHA using the primers pB-aprEHA-F/R. The amplicon was further fused to the plasmid pBsacA using primers pB-aprEHA-b-F/R, producing pBsacA-aprEHA. The gene of mCherry was amplified from the plasmid pBP43-GFP-mCherry using primers pB-mCherry-F/R prior to fusing with the linearized plasmid pBsacA-aprEHA (amplified by primers pB-mCherry-b-F/R) by Gibson assembly, generating pBsacA-aprEHA-mCh. As Golden gate assembly requires two *Bsa*I restriction site and orthogonal overhangs to ensure the target fragment correctly being inserted into the recipient vector, we used pBsacA-aprEHA-mCh as a template and perform two rounds of rPCR to obtain two *Bsa*I restriction sites using primers *Bsa*I-1-F/R and *Bsa*I-2-F/R, so as to enable the resulting plasmid being digested by *Bsa*I. The crRNA sequence targeting *aprE* was amplified from pHTaprE using primers aprEcrRNA-*Bsa*I-F/R. To construct pB-sacAKo-aprEKi, a restriction-ligation was performed in a mixture containing crRNA expression cassette targeting *aprE*, the recipient vector pBsacA-aprEHA-mCh, *Bsa*I enzyme and T4 DNA ligase, generating pB-sacAKo-aprEKi. For double deletion of *sacA* and *aprE*, a single crRNA array was synthesized from GENEWIZ, Inc. (Suzhou, China), which contained P_vegM_ promoter, crRNA targeting *sacA* and *aprE*, as well as BT5 terminator (screened by our laboratory). The array was amplified from pUC57 plasmid using primers SA-BT5-F/R, the resulting PCR product was inserted into pB-sacAHA-aprEHA plasmid (containing the homologous arms of *sacA* and *aprE*) with primers SA-BT5-b-F/R by Gibson Assembly, yielding the recombinant plasmid pB-PvegM-SAKo.

### Plasmid Curing

To cure mutant strains of the pBSG so as to enable their use in a second round of genome editing, the mutant strains were inoculated into LB medium with a final concentration of 0.0005% SDS without antibiotics (Trevors, 1986), and then incubated at 37°C, 200 rpm for 20 h. Accordingly, the culture was diluted and spread on LB plates without antibiotics. Colonies were carefully picked up and dotted at the same positions on two LB plates with and without antibiotics, respectively. Antibiotics-sensitive colonies were picked and propagated in 5-mL LB medium. Then, plasmid-free mutants were further confirmed through PCR. For elimination of pHT01 and pAD123 plasmid, we referred to previous research and achieved it (Yamashiro et al., 2011; So et al., 2017).

## Results and Discussion

### Design of a CRISPR-Cpf1 in All-in-One (AIO) System for Deletion of Large Genes in *B. subtilis*

**FIGURE 2:**
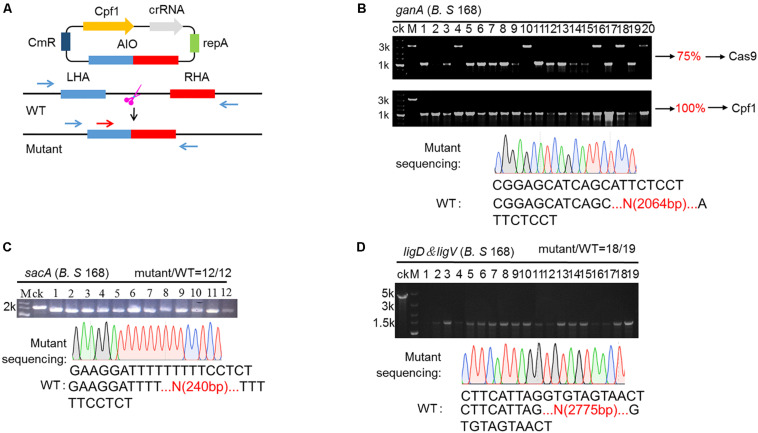
CRISPR-Cpf1-mediated genome editing in the *Bacillus subtilis* via using AIO system. **(A)** Schematic illustration of the editing procedures. The blue arrows are the primers utilized for PCR validation of the editing efficiency. The red arrow is the primer used for sequencing. **(B)** Deletion of *ganA* gene mediated by AIO system based on CRISPR-Cpf1 and CRISPR-Cas9 in *B. subtilis* 168. The efficiency of deletion of *ganA* by AIO system based on CRISPR-Cpf1 was 20/20 (100%). The efficiency of deletion of *ganA* gene by AIO system based on CRISPR-Cas9 was 15/20 (75%). Lane M, the 5-k DNA marker from Takara with number on the left representing the band size in kb. The lane labeled “ck” is the PCR product from the wild-type strain as a control. **(C)** AIO-mediated deletion of the *sacA* gene in the *B. subtilis* 168 strain. The editing efficiency was 12/12. **(D)** AIO-mediated deletion of the *ligD* & *ligV* genes in the *B. subtilis* 168 strain. The editing efficiency was 18/19.

To achieve the goal for highly efficient editing the genome in *B. subtilis*, we employed a type V Cas protein, Cpf1 from *Francisella novicida* to design an all-in-one system (AIO). In this system, crRNA and Cpf1 were expressed constitutively by P_veg_ and P43 promoters, respectively. The expressed crRNA carried Cpf1 to a specific site of the target gene, where the cleavage of Cpf1 to the target gene would cause the precise recombination of homologous segments, leading to the deletion of the target gene (Figure 2A). To compare the performance of gene editing between Cpf1 and Cas9, we constructed a CRISPR-Cas9-based AIO system. To verify the functionality of two AIO systems, we selected the *ganA* gene (gene of medium size) to identify the efficiency of deletion mediated by the two AIO systems. The *ganA*, which was of 2064 bp in length, encodes β-galactosidase, which participates in the degradation of galactose. Disruption or complete deletion of *ganA* does not affect the growth of host. Therefore, we chose *ganA* gene as the target to explore the functionality of the two designed CRISPR systems (CRISPR-Cpf1 and CRISPR-Cas9) for deletion of large genes. The two editing plasmids, pHTganA-Cpf1 (refer to addgene #158647) and pHTganA-Cas9, were separately introduced into *B. subtilis* 168. The transformants were separately picked up and then inoculated into newly prepared LB medium to grow. Then the culture was diluted to appropriate density and spread onto LB agar plates. Twenty clones were randomly picked for cPCR to screen *ganA-*disrupted mutants from pHTganA-Cpf1 and pHTganA-Cas9 plates, respectively. The results showed that 20 clones of *ganA*-disrupted mutants had smaller cPCR product than that of wild-type strain (ck), indicating that *ganA* has been successfully deleted on pHTganA-Cpf1 plate (efficiency was 100%, Figure 2B). However, we only screened 15 *ganA*-deletion mutants on the pHTganA-Cas9 plate (efficiency was 75%, Figure 2B). Furthermore, the precision of deletion sites for these engineered strains were validated by sequencing the cPCR products (Figure 2B). These data displayed that the efficiency of CRISPR-Cpf1 and CRISPR-Cas9 systems was sufficient for general gene editing. However, CRISPR-Cpf1 system would be more suitable for larger fragments to be knocked out. Therefore, our following experiments all aimed at the engineering design of CRISPR-Cpf1 system. To further verify the functionality of the AIO system based on CRISPR-Cpf1, we selected *sacA* to identify the efficiency of deletion mediated by the system. The *sacA* (NC_000964:3902858), which was of 2400 bp in length, encodes sucrose-6-phosphate hydrolase, which is not an essential gene for *B. subtilis* (Zhu and Stulke, 2018). In most integrated systems for *B. subtilis*, *sacA* site was widely used to an integration site (Radeck et al., 2013). Here, we selected PAM sequence 5′-TTTG-3′ (Zetsche et al., 2015) in *sacA* gene, and then constructed PHTsacA plasmid harboring *Cpf1* gene and target-*sacA* crRNA that under the control of constitutive promoters P43 and P_veg_, respectively (Figure 2A). The 1200-bp homologous arm was inserted downstream of the CRISPR-Cpf1 expression cassette. Based on the deletion design, the fragment should be 240 bp after deletion. Plasmid pHTsacA was then introduced into *B*. *subtilis* 168. The transformations were picked, inoculated into 5 mL of LB medium, and incubated aerobically at 37°C overnight. Then the culture was spread onto LB plates. Twelve colonies were randomly collected to perform colony PCR (cPCR) to verify the deletion efficiency. The PCR product should be of 1655 bp if *sacA*-deletion was unsuccessful using designed primers, in contrast, it should be of 240 bp smaller than that of the wild type *B. subtilis* 168. The data showed that introduction of pHTsacA into *B. subtilis* 168 resulted in 100% deletion (Figure 2C). These results were further confirmed by sequencing of one successfully deleted mutant (Figure 2C), indicating that the designed synthetic CRISPR-Cpf1 tool has relatively high editing efficiency targeting genes with short lengths.

Furthermore, to further identify the available range of AIO-based CRISPR-Cpf1 system, we employed the system to delete larger gene cluster. The *ligD* & *ligV* gene cluster, which is of 2775 bp in length, was selected as the target. The gene cluster *ligD* & *ligV* is involving in the non-homologous end-joining (NHEJ) process (Pitcher et al., 2007). Although the gene cluster is important in maintaining chromosomal stability in bacteria, it is non-fatal to *B. subtilis* when *ligD* & *ligV* cluster is deficient. Therefore, we chose *ligD* & *ligV* gene cluster as the target to test the functionality of deletion of large gene cluster using designed CRISPR-Cpf1 system. The AIO system was selected to construct the editing system (Figure 2A). The pHTDV plasmid harboring *ligD & ligV*-targeting crRNA and the homologous arms was constructed in the similar procedure as that of pHTganA but had longer homologous arms (600 bp). The editing plasmid, pHTDV, was introduced into *B. subtilis* 168. The transformants were picked and then inoculated into newly prepared LB medium to grow. Then the culture was diluted to appropriate density and spread onto LB agar plates. Nineteen colonies were randomly picked up to screen *ligD* & *ligV-*disrupted mutants by cPCR. The results showed that 18 of the 19 clones were confirmed to be the *ligD* & *ligV-*deficient strains, suggesting an editing efficiency of 94.7% (Figure 2D). Furthermore, the precision of deletion sites for these engineered strains were validated by sequencing the cPCR products. The data displayed that all these *ganA*, *sacA* and *ligD* & *ligV* mutants had accurate deletion sites as we had designed (Figures 2B–D).

In this work, we authenticated that the AIO system was capable of being applied to delete single gene with high efficiency in *B. subtilis*. AIO-based gene editing employing CRISPR-Cas9 was also constructed in *B. subtilis* ATCC 6051a (Zhang et al., 2016). The prominent feature of AIO was that Cas protein, sgRNA and homologous arms were all concentrated on one plasmid. When the gene editing was completed, the plasmid will be eliminated and no foreign genes will be introduced into the genome. However, the plasmids of AIO were often large, which led to low transformation efficiency and instability of plasmids (Supplementary Table S5). To improve the stability of plasmid replication, we use pHT01 vector as the skeleton of AIO, which carries a repA replicon. The vector pHT01 belongs to a medium copy plasmid. Because repA protein in the way of θ replication to multiply, the pHT01 vector is relatively stable. If we want to further improve the stability of the plasmid, we can integrate Cpf1 into the host genome, which can reduce the replication pressure of plasmid. Therefore, to break through the limitations of AIO system and provide flexible tools to facilitate gene editing in complex genetic context, more robustness, and efficiency of CRISPR-Cpf1 system should be built to allow reliable genome editing.

### Construction and Validation of Two-Plasmid (TP)-Based, Cpf1-Mediated Gene-Deleting System in *B. subtilis*

**FIGURE 3:**
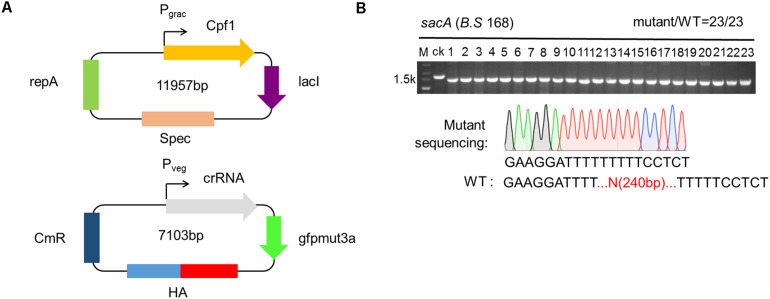
Construction of the two plasmids (TPs) for genome engineering system. **(A)** Schematic illustration of pHTCpf1 plasmid and pADsacA plasmid containing *sacA* crRNA transcription module as well as donor DNA template. **(B)** TPs-mediated disruption of the *sacA* gene in the *Bacillus subtilis 168* strain. The editing efficiency was 20/20. The lane labeled “ck” is the PCR product from the wild-type strain as a control.

To broaden an alternative form of CRISPR-Cpf1 system, the two plasmids (TP) system, and verify whether the system was able to efficiently delete genes in *B. subtilis*. In this system, we also chose *sacA* as the target. First, we constructed plasmid pHT01-Cpf1 (refer to addgene #158648), harboring Cpf1 under the control of the inducible promoter P_grac_ (Figure 3A). Because pHT01 and pAD123 contained the same chloramphenicol resistance gene, we substituted the chloramphenicol-resistant gene from pHT01 vector with spectinomycin-resistant gene, allowing to screen the transformants with different antibiotics (Figure 3A). We constructed plasmid pADsacA (refer to addgene #158649), derived from the backbone of pAD123, to constitutively express the crRNA targeting *sacA* mediated by P_veg_ promoter (Figure 3A). Then, we sought to identify the efficiency of disruption of *sacA* by TPs system. We sequentially transformed plasmids pHT01-Cpf1 and pAD-sacA into *B. subtilis*. The two types of transformants were picked up, and inoculated into LB medium to propagation. When OD_600_ reached approximately 0.5, Isopropyl-β-D-thiogalactopyranoside (IPTG) with final concentration of 1 mM was added to induce the expression of Cpf1. Then the culture was spread onto LB agar plates. After incubated overnight, 23 colonies were randomly picked to perform cPCR to verify the deletion efficiency. The results exhibit that all the colonies (23/23) were the successfully deleted mutant harboring a 240 bp-deleted sequence of *sacA* (Figure 3B). To further validate the accuracy of gene deletion, we confirmed the sequence of disrupted *sacA* by sequencing. The data validated the successful deletion of 240 bp of *sacA*, suggesting that the deletion efficiency mediated by TPs strategy is as high as 100% (Figure 3B). To further compare the performance CRISPR-Cpf1 and CRISPR-Cas9 using two-plasmid strategy to knock out the same gene in *B. subtilis* 168, we selected *ganA* (2064 bp) gene as our target. The results showed that the knockout efficiency of CRISPR-Cpf1 system was much higher than that of CRISPR-Cas9 system (10/11 vs. 6/11, Supplementary Figure S2). These data suggest that the function of CRISPR-Cpf1 in knockout of single gene is higher than that of CRISPR-Cas9 with the same strategy (two-plasmid system).

### CRISPR-Cpf1-Based CIGE (CCB-CIGE) Platform Is Superior to Highly Efficient Gene Insertion in *B. subtilis*

**FIGURE 4:**
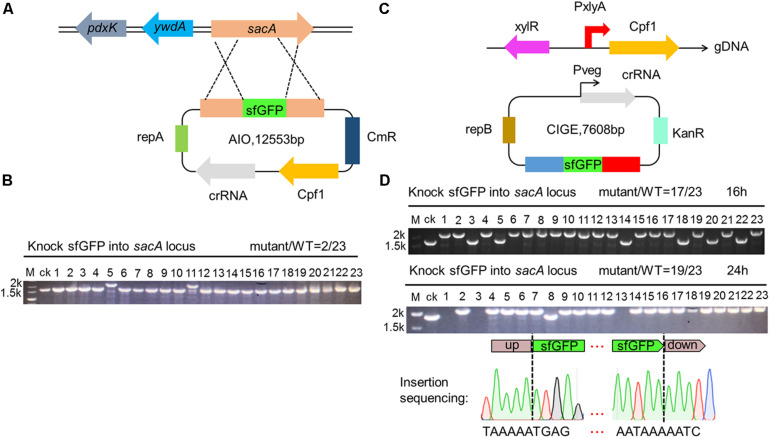
Construction of superfolder green fluorescence protein (sfGFP) insertion in *sacA*. **(A)** Scheme showing the procedures for gene insertion into *Bacillus subtilis* 168 by AIO system. **(B)** cPCR results shown that 9% (lane 5 and lane 11) of colonies had the sfGFP insertion mutant. **(C)** Scheme showing the procedures for gene insertion into *B. subtilis* 168 by CCB-CIGE system. **(D)** CIGE enables highly efficient sfGFP insertion mutation in the *B. subtilis* 168 strain. The *sacA* gene was replaced by the *sfgfp* gene. The efficiency for *sfgfp* gene insertion was 17/23 in the *B. subtilis* 168 strain. Prolonged incubation time under selective pressure increased the mutation efficiency to 82%.

Gene insertion is another critical issue to genome editing. Therefore, robust and efficient insertion system is valuable tool for precisely editing the genome of *B. subtilis*. To evaluate the availability of the CRISPR-Cpf1-based AIO system for insertion of heterologous gene into the genome of *B. subtilis*, we firstly constructed an AIO plasmid pHTsfGFPKi, of which the expression of Cpf1 and *sacA*-targeting crRNA was controlled by P43 and P_veg_, respectively. The coding region of *sfGFP* was flanked by upstream and downstream homologous arms of *sacA* (Figure 4A). We introduced pHTsfGFPKi into *B. subtilis* 168 to implement insertion function. We randomly chose 23 colonies from the plate and performed cPCR. By evaluating the size of cPCR product for each selected mutant, we found that two clones contained larger size of cPCR product than that of the “ck,” whereas the others had the same size as that of the ck, indicating that *sfGFP* was successfully inserted into the genome of the two clones among the 23 selected colonies. The efficiency of insertion was calculated to be 9% (2/23) (Figure 4B). We infer that because the expression of Cpf1 or crRNA might be unstable from the plasmid that is too large in size.

To resolve the problem of low insertion efficiency by AIO system, we designed CRISPR-Cpf1-based CIGE (CCB-CIGE) platform to improve the expression of the components to elevate efficiency of gene insertion (Figure 4C). In this strategy, we chose pBSG as the skeleton and constructed a plasmid, pBsfGFPKi, harboring a *sacA*-targeted crRNA sequence controlled by P_veg_. The coding region of *sfGFP* was flanked by upstream and downstream homologous arms. Because pBSG vector belongs to high copy plasmid, we can improve the expression of each component by using it. However, the replication protein of repB carried by the plasmid will multiply in the form of rolling circle replication, which may lead to instability of the plasmid. The plasmid was then introduced into the host BS-*ganA*’-Cpf1 (the Cpf1 gene under the control of P_xylA_ was integrated in the chromosome of *B. subtilis* via substitution for *ganA*). After 16 h induction by 1% xylose, 23 colonies were randomly chosen to identify the insertion results by cPCR. The data showed that the cPCR products from 17 out of 23 clones were larger compared to that of ck, indicating that the *sfGFP* had been successfully inserted into the chromosome of these clones (Figure 4D). The insertion efficiency was confirmed to be 74% (Figure 4D). According to the previous research, efficiency of gene knock-in in *B. subtilis* can be significantly improved by iterative genome engineering (So et al., 2017). Therefore, we prolonged the incubation time to 24 h, and the insertion efficiency increased to 82% (Figure 4D). To further validate that insertion occurred precisely at the *sacA* site, we sequenced the region from the upstream to the downstream homologous arm of No. 1 mutant among the successfully-inserted mutants. The map of sequencing revealed that *sfGFP* has been accurately inserted into specific site of the *sacA* gene using an optimized CRISPR-Cpf1-mediated gene knock-in strategy, without any unintended mutations (Figure 4D). These results manifest that our optimized CRISPR-Cpf1 system accurately insert target genes into preset positions in the chromosome.

Gene knock-in based on CRISPR system is an important technology for metabolic engineering and genomic function research. A potential application is that when we construct metabolic engineering strains, we often need to overexpress some genes to improve the titer of metabolites. However, overexpression of some genes on plasmids often brings great pressure to the host. Therefore, integrating the target gene into the genome for overexpression has become a preferred strategy. Another advantage is to study the function of some genes in the genome. When we study a gene in the genome, we often take the surrounding genetic environment into account. At this time, *in situ* expression of gene is often much more important than overexpression on plasmid. Although we have constructed a gene knock-in system based on CRISPR-Cpf1 in this paper, we only verified it using *sf*GFP. In the current research, large gene cluster knock-in is an indispensable technology for the study of synthetic biology. Therefore, the strategy of gene knock in based on CRISPR-Cpf1 constructed in this work needs to be further optimized and the ability of large gene cluster knock-in needs to be improved. As the CCB-CIGE strategy was more efficient than AIO, we used CCB-CIGE in the following experiments.

### Design of CRISPR-Cpf1 for Precise Deletion of Large Gene Cluster

Previous studies have shown that the deletion of large genome in *Bacillus* sp. plays a crucial role in heterologous expression of proteins, genome reduction (Westers et al., 2003), strain improvement (Thwaite et al., 2002) and overproduction of antibiotics (Zobel et al., 2015). There are three large gene clusters in *B. subtilis* encoding polyketide synthase (*pks*), plipastatin synthetase (*pps*), and surfactin (*srf*), which account for 7.7% of the total genome (So et al., 2017). There are also gene clusters in *B. subtilis* that synthesize peptide antibiotics, such as *bac* operon (Ozcengiz and Ogulur, 2015). The deletion of these gene clusters that synthesize secondary metabolites has minor effect on the growth of *B. subtilis*. Moreover, deletion of these non-essential regions in *B. subtilis* is essential to construction of the minimal genome in microbial chassis. Although a counter-selectable marker system based on synthetic gene circuits has been developed to delete *pps* operon, the knockout efficiency was only 6.4% (Jeong et al., 2015). Recently, a new editing system based on CRISPR-Cas9 employing a single sgRNA was developed to delete *pps* operon (So et al., 2017). However, the *pps* operon was not completely deleted even though the efficiency of deletion was further improved by optimizing. We infer that the DSB site and the repair site of homologous arm are too far to initiate an efficient double cross-over.

**FIGURE 5:**
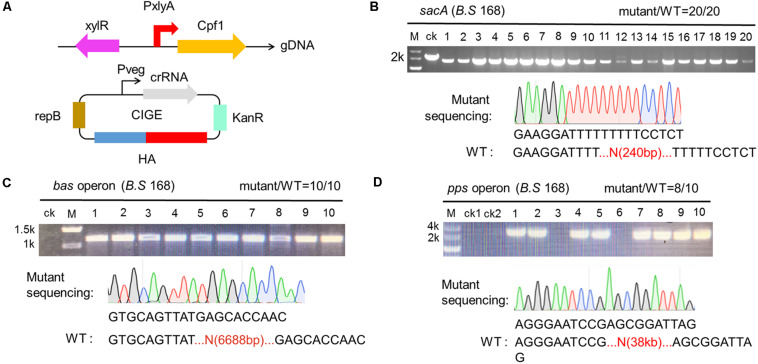
Deletion of larger gene clusters mediated by CCB-CIGE system derived from CRISPR-Cpf1. **(A)** Scheme showing the composition of the CIGE system for the deletion of larger gene clusters. **(B)** CIGE-mediated disruption of the *sacA* gene in the *Bacillus subtilis* 168 strain. The editing efficiency was 20/20. **(C)** Identification of *bac* operon deletion mutants. *bac* operon is composed of seven genes (*bac*ABCDEFG), about 6688 bp. Ten transformants were selected and verified by colony PCR. M and ck represent the 2-kb DNA ladder and wild-type *B. subtilis* 168, respectively. **(D)** Confirmation of *pps* operon deletion using colony PCR. Lane M, the 5-kb DNA marker from Takara. Lane ck1 and ck2, PCR amplification with the *B. subtilis* 168 and *B. subtilis* comK genomic DNA (gDNA) using external primers, respectively. Lane 1-10, PCR amplification with the mutant gDNA using external primer. The editing efficiency was 8/10.

In view of those results that CIGE-based CRISPR-Cpf1 system was highly functioned to insertion of gene in chromosome, we considered that whether the system was also suitable to deletion of large gene clusters in *B. subtilis*. To verify the deduction, we first evaluated the function of deletion of *sacA* (Figure 5A). Plasmid pBsacA (refer to addgene #158650), containing *sacA*-targeted crRNA and the corresponding homologous arms identical to that of AIO and TPs (refer to pHTsacA), was constructed to implement the function. Then, pBsacA was introduced into BS-*ganA*’-Cpf1. We randomly selected 20 colonies from the agar plate and perform cPCR to identify the deletion efficiency. The results demonstrated that all the colonies harbored the disrupted *sacA* smaller than that of the wild-type *sacA* (ck), suggesting that the genome editing efficiencies mediated by CIGE system was 100% (20/20) (Figure 5B, upper panel). Sequencing results also authenticated that *sacA* has been disrupted by 240 bp in the CIGE edited hosts (Figure 5B, lower panel).

After validating that *sacA* was deleted by the engineering CRISPR-Cpf1 system, we sought to further explore whether the system can be used to delete larger gene clusters. The *bac* operon was selected as the deletion target. Firstly, we constructed the targeting plasmid, pBbac, containing expression cassette composed of a pair of 500-bp homologous arms and a *bacD*-targeted crRNA under the control of P_veg_. After transformation in BS-*ganA*’-Cpf1, the transformants harboring pBbac was cultured and screened in line with the procedure above. Since the designed primers for verification flanked the homologous arms, the product of cPCR should be approximate of 1320 bp rather than 8008 bp (the full-length of *bac* operon) if *bac* operon was successfully deleted. We randomly selected 10 colonies from the agar plate and performed cPCR to identify the deletion efficiency. Interestingly, the cPCR product from the 10 colonies had expected gene size while the control (ck) hadn’t, indicating that *bacD* was successfully deleted from the operon (Figure 5C). In parallel, we sequenced the cPCR product from one of the successfully deleted mutants and confirmed the deletion (Figure 5C).

To testify whether the tool functioned to a broad range of targets, we selected another target, the *pps* operon, to evaluate the deletion efficiency. We firstly verified that the *pps* operon exists in the *B. subtilis* 168 prior to performing the deletion of the *pps* operon (Supplementary Figure S1). Then, we replaced *bacD*-targeting crRNA with *ppsC*-targeting crRNA on plasmid pBbac, and extended the length of homologous arms to 800 bp to ensure the deletion efficiency. Plasmid used to delete *pps* was termed pBpps. Then, pBpps was transformed into BS-*ganA*’-Cpf1. The screening and verification procedure were identical to that of *bacD* deletion. The cPCR product from *ppsC*-deleted mutant should be of 1772 bp by the designed primers. The results of cPCR showed that 8 out of 10 colonies had the products of expected size, suggesting that *ppsC* is deleted in these 8 mutants (Figure 5D). These results were further confirmed by sequencing (Figure 5D). The deletion efficiency for *bac* and *pps* was of 100% and 80%, respectively, based on the CRISPR-Cpf1-dependent CIGE platform (Figures 5C,D).

These results manifest that our designed CRISPR-Cpf1 system (CIGE) is more reliable and portable than the CRISPR-Cas9 system, especially for deletion of large gene clusters. To our best knowledge, this is the most efficient system to delete a *pps* operon with a single crRNA (Figure 5D). Deletion of large gene fragments is very important in synthetic biology, especially in deleting non-essential genes to construct minimal genomic microorganisms. Therefore, our customized CRISPR-Cpf1 system has great potential to implement this.

### CCB-CIGE Platform Is Upgraded to a Higher Version to Exert Multiplex-Gene Editing

Wild-type bacteria usually are unable to exert programmed functions preset in cell factories. Therefore, engineering natural bacteria is a practical strategy to design a higher version of microbial chassis for synthetic biology. Precise and portable multiplex gene editing is an absolutely indispensable approach to do this. However, some commonly used methods, Cre/*lox*P (Suzuki et al., 2005; Choi et al., 2015) and *Red* recombination system (Datsenko and Wanner, 2000), were difficult to efficiently edit at genome-scale because of some technical hurdles. First of all, Cre/*lox*P system is not a scarless knockout technology, which leaves loxPLR sites at the edited position (Suzuki et al., 2007). These scars may affect the growth of host cells, and the cycle of this technique is longer. Secondly, the efficiency of this gene deletion method (Cre/*lox*P) is not very efficient because two rounds of homologous recombination are required and mutant selection after the second recombination is time-consuming (Cleto et al., 2016). Thirdly, it is difficult to achieve simultaneous editing of multiple targets in the genome editing system based on Cre/*lox*P. For genome editing in prokaryotes, phage-derived lambda red recombinases have been employed in recombineering, which facilitates homology-dependent integration/replacement of a donor DNA or oligonucleotide. However, these systems require the genetic background of the target strains such as deficiency of methyl-directed mismatch repair or *RecA* that involves in the recombinational DNA repair system (Wang et al., 2009). Multiplex genome editing systems based on CRISPR-Cas9 have been applied to different organisms, including *E. coli* (Zerbini et al., 2017), *B. subtilis* (Westbrook et al., 2016), *Streptomyces* species (Cobb et al., 2015), *Rhodosporidium toruloides* (Otoupal et al., 2019), and mammalian (Cong et al., 2013).

To fully exploit the function of CRISPR-Cpf1 in multiplex genome editing, we employed two genes used above, *sacA* and *aprE*, to design and build a multiple gene editing system. Firstly, we incorporated *sacA-* and *aprE-* crRNA cassettes into pBSG, by which the two crRNAs can simultaneously target *sacA* and *aprE*. In this form, we found when the crRNAs were transformed into BS-*ganA*’-Cpf1, only one gene was deleted in the same transformant (data not shown). Previous study reported that *B. subtilis* has more complex recombination systems and diverse plasmid replication modes. Therefore, we inferred that the failure might be due to the exchange of some fragments of the plasmid in the process of replication. After sequencing the transformants with single gene deletion, we confirmed that the sacA crRNA expression cassette was lost from plasmid, resulting in failure of deleting *sacA* (data not shown).

**FIGURE 6:**
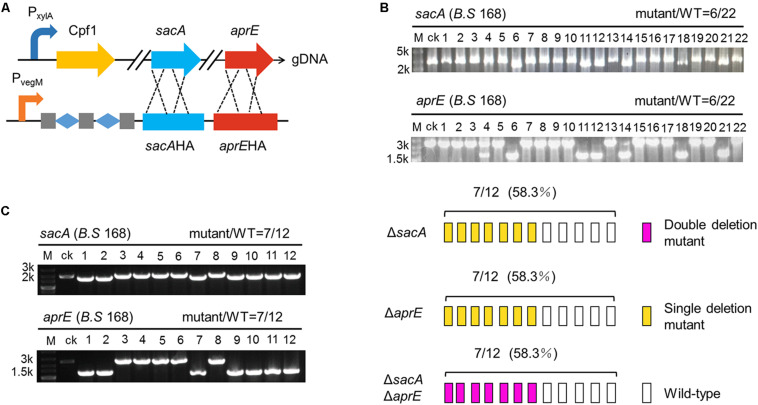
CRISPR-Cpf1-assisted simultaneous deletion of double genes (ΔsacAΔaprE). **(A)** The construction of the Cpf1 multiplex gene editing system contains a Cpf1 expression cassette and a multi-crRNA expression cassette. Cpf1 was inserted *ganA* site of genome DNA (gDNA) of *Bacillus subtilis* 168. The multi-crRNA expression cassette contains three DR guide units, and each unit includes one mature DR and 23 bp of guide sequence. **(B)** Multi-crRNA expression cassette is controlled by the P_veg_ promoter. Twenty-two colonies were picked and screened for mutations. The efficiency of simultaneous deletion of double genes is 6/22. **(C)** Multi-crRNA expression cassette is controlled by the P_veg_ promoter variant P_vegM_. The colonies of 7/12 is identified as a double deletion mutant strain (ΔsacAΔaprE). The open rectangle, orange rectangle, and dark pink rectangle represent the WT strain, the single deletion mutant of ΔsacA or ΔaprE, and the ΔsacAΔaprE double deletion mutant, respectively.

According to previous studies, Cpf1 intrinsically processes pre-crRNA into mature crRNA by cleaving specific site (Fonfara et al., 2016; Zetsche et al., 2017). Thus, we inferred that it might be feasible to implement multiplex genome editing by integrating multiple crRNAs to single expression cassette. Two crRNAs targeting *sacA* and *aprE* were genetically fused and insulated them by synthetic two repeats of “Direct Repeat (DR)-Spacer” units. The homologous arms of *sacA* and *aprE* tandem were sequentially cloned downstream of these crRNAs, generating a complete targeting sequence. P_veg_ was equipped to trigger the expression cassette. The expression cassette was then cloned into pBSG, yielding pB-PvegW-SAKo (Figure 6A). pB-PvegW-SAKo was transformed into BS-*ganA*’-Cpf1. Through the cultivation and screening as described above, cPCR results showed that 27.2% (6/22) of colonies were *sacA*- and *aprE-*deficient (Figure 6B). Although we successfully achieved double-gene deletion by CCB-CIGE platform, the efficiency still needs to be improved so as to elevate the work performance.

Accordingly, the next thrust is to engineer the components on this platform. Because the wild type P_veg_ promoter is a super strong promoter, we deduced that excessive activity of P_veg_ in the cell might induce growth burden in *B. subtilis*, rendering low editing efficiency through disturbing expression of crRNAs. Therefore, to rebalance the expression, we mutated the -10 region of the wild-type P_veg_ promoter to decrease its activity, variant was termed P_vegM_ (Figure 6A). By substitution for the wild-type P_veg_, pB-PvegM-SAKo was constructed, and then transformed into BS-*ganA*’-Cpf1. We randomly chose 12 colonies to verify the deletion by cPCR. The results showed that 7 selected clones among the 12 clones harbored the deleted *sacA* and *aprE* genome as the bands were smaller than that of the control “ck” (Figure 6C). The double-deficient mutants accounted for 58.3% of total tested mutants (Figure 6C), which was higher than that of previously reported by two folds (Figures 6B,C). These results suggest that the optimized CRISPR-Cpf1 system has great potential to achieve multiplex-gene editing at genome-scale.

Up to date, CRISPR-Cas9 system has been widely used in different organisms for genome editing. However, the function relies on a complex composed of crRNA and tracrRNA (or a chimera gRNA), which guides Cas9 to the target in genome. In contrast, one single crRNA is sufficient to guide CRISPR-Cpf1 RNP to a gene target. Practically, CRISPR-Cas9 system mediates multiplex genome editing by expression of multiple gRNAs. Nevertheless, the functional construct is often relatively large and complex, which would be rather difficult to construct and transform plasmid. Compared with Cas9 nucleases, the most significant feature of Cpf1 nucleases is that they not only have DNase activity but also RNase activity, which gives them the ability to process their own crRNA from a long precursor. This feature greatly facilitates their application for multiplex genome editing, transcriptional regulation and imaging, which tasks typically need to locate multiple loci in the genome for efficient operation. By taking advantage of this feature, we successfully constructed the multiplex genome editing system by using a single crRNA array in one vector (Figure 6A). The potential advantage of this system is that it can delete those genes that are difficult to delete individually. Another consideration is that the transformation efficiency of multiplex-gene-targeting plasmid is significantly lower than that of single-gene-targeting plasmid. It might also be due to the strong cleavage effect of crRNA by the constitutive transcription of the P_veg_ promoter, which makes it difficult for bacteria to repair in time. Therefore, inducible promoters can be used to regulate the expression of crRNA to enrich biomass in later studies.

Up to now, we have constructed a set of CRISPR-Cpf1-based toolkit (including AIO, TP, and CCB-CIGE) in *B. subtilis*. Zhang et al., Westbrook et al., and Wu et al., have developed genome editing tools based on CRISPR-Cas9 and CRISPR-Cpf1 in previous studies. However, compared with their systems, our newly constructed system has some advantages.

Firstly, although Zhang et al., constructed a genome editing system based on CRISPR-Cas9 in *B. subtilis* ATCC 6051a, they only verified that CRISPR-Cas9 system can disrupt gene in *B. subtilis* ATCC 6051a, and did not testify that the system can accurately delete and insert gene in the frame. In this work, we constructed a genome editing system based on CRISPR-Cpf1 with the same strategy (AIO). In this system, we used strong promoters P43 and P_veg_ from *B. subtilis* to express Cpf1 and crRNA, respectively. And we also verified that our engineered AIO system is capable of knocking out fragments of different sizes (240 bp, 2064 bp and 2775 bp) with efficiency over 95% (Figures 2B–D). Simultaneously, we construct two AIO systems (based on CRISPR-Cpf1 and CRISPR-Cas9) to prove that CRISPR-Cpf1 system is superior to CRISPR-Cas9 system in *B. subtilis* (take gene *ganA* as an example, 100% of Cpf1 vs. 75% of Cas9, Figure 2B). However, the AIO system we constructed in this study is relatively larger, which can make the construction and transformation of plasmid a little more difficult.

Secondly, in the study of Westbrook et al. (2016) they constructed a tool kit based on CRISPR-Cas9 in *B. subtilis*. In their whole research, they all adopt chromosome integration strategy to achieve different functions (including knockout, knock-in and transcription interference) based on CRISPR-Cas9. In this way, foreign genes (for example, Cas9, gRNA, and resistance marker) will inevitably be introduced into the genome of *B. subtilis*. However, as an important platform for metabolic engineering and synthetic biology research, the effect of exogenous gene introduction on the expected function of *B. subtilis* is unknown. Concurrently, insertion of antibiotic resistance marker will disqualify the use of engineered *Bacillus* strains as an eco-friendly host for food-grade applications, industrial fermentation and bioremediation. Westbrook et al. (2016) have also constructed a multi-gRNA delivery vector for multiplex genome editing. However, it is relatively difficult and time-consuming to insert multiple repeat sequences into one vector at the same time. In this study, we constructed a toolkit based on CRISPR-Cpf1, which can be used in different scenarios. AIO and TP systems can be used for traceless knockout, and are suitable for use without introducing foreign gene (for example, fermentation of food-grade enzymes). CCB-CIGE system can be used for engineering modification of *B. subtilis* to produce industrial enzyme. Recently, Wu et al., developed a toolkit (including knockout, knock-in, point mutation and transcription interference and activation) based on CRISPR-Cpf1 in *B. subtilis*. However, they did not explore the editing efficiency of a single gene of CRISPR-Cpf1 system in *B. subtilis*. It is unclear whether the editing efficiency varied in deletion of different size of the fragment. In our study, the AIO system we developed can knock-out small and medium-sized fragment, and the knock-out efficiency is almost not affected (efficiency is between 95% and 100%, Figures 2B–D). And in previous studies, it has been shown that the knockout of non-essential large gene clusters is indispensable for the construction of the minimal genomic chassis microorganism. However, in the study of Wu et al., deletion of large gene clusters was not validated. Here, we developed an upgraded CRISPR-Cpf1 system, which can knock-out *bac* (efficiency is 100%) and *pps* operons (efficiency is 80%, Figures 5C,D). And as far as we know, this is the first example to knock out 38-kb gene cluster with CRISPR-Cpf1 system in *B. subtilis*. Next, we also try to use CCB-CIGE platform to delete extremely large cluster *pks* operon (about 78 kb). However, we only obtain lower deletion efficiency (about 16.7%, data not show). Although, we still believe that the optimized CCB-CIGE platform can continue to improve the knock-out efficiency of *pks* operon. For double gene editing, Wu et al., used a double plasmid expression system, and enhanced the efficiency of homologous recombination by overexpression of mutated NgAgo^∗^. However, they need to add two inducers to achieve the goal of knocking out two genes, which is more laborious. Moreover, the transformation efficiency of the double plasmid system is significantly lower than that of the CCB-CIGE system in our study. Similarly, in the research of Jiang et al., they also overexpressed the recombinant factor *recT* to improve the efficiency of recombination. However, in our study, Cpf1 is integrated into the chromosome, the decrease of Cpf1 expression may weaken the knockout efficiency. And we did not introduce the factor NgAgo^∗^ and recT, to enhance homologous recombination, which will also reduce the editing efficiency. It is necessary to continue to improve the efficiency of homologous recombination and multi-target editing of our system.

Overall, we identified that engineering CRISPR-Cpf1 system significantly facilitates gene manipulation in *B. subtilis*. The development of this system has accelerated the construction of high-version microbial chassis. We believe that the optimized CRISPR-Cpf1 system can also be applied to other Gram-positive bacteria, such as *Bacillus thuringiensis* and *Bacillus licheniformis*.

## Data Availability Statement

All datasets generated for this study are included in the article/Supplementary Material.

## Author Contributions

WH and WC conceived the project and designed the experiments. FS performed the molecular cloning experiments. QL and QC performed the colony PCR. WH, WC, and ZZ analyzed the data and wrote the manuscript. All authors contributed to the article and approved the submitted version.

## Conflict of Interest

The authors declare that the research was conducted in the absence of any commercial or financial relationships that could be construed as a potential conflict of interest.
