# Supplementary material for: Design and Construction of Portable CRISPR-Cpf1-Mediated Genome Editing in Bacillus subtilis 168 Oriented Toward Multiple Utilities
Source: Front Bioeng Biotechnol. 2020 Sep 2;8:524676. doi: 10.3389/fbioe.2020.524676 (PMC7492563; doi:10.3389/fbioe.2020.524676)
Supplement: Supplementary file 1 [file Data_Sheet_1.docx]

Supplementary Material

**Supplementary Table 1. Plasmids and strains used in this study.**

| **Plasmids and strains** | **Relevant characteristics** | **References or source** |
| --- | --- | --- |
| Plasmids |  |  |
| pAX01 | *B. Subtilis* integration vector，xyl^r^-P_xyl_ cassette，*spec*^r^，*Cm*^r^ | Lab stock |
| pAX-Cpf1 | Derived from pAX01, *Cpf1* inserted | This study |
| pHT01 | IPTG-inducible expression vector for *B.subtilis* carrying P_grac_ and *Cm*^r^ | Lab stock |
| pHTsacA | pHT01 derivative，containging *Cpf1* expression cassette，*sacA* targeting 23bp crRNA transcription module and 1.2kb donor DNA | This study |
| pHTganA-Cpf1 | pHT01 derivative，containging *Cpf1* expression cassette，*ganA* targeting 23bp crRNA transcription module and 1kb donor DNA | This study |
| pHTganA-Cas9 | pHT01 derivative，containging *Cas9* expression cassette，*ganA* targeting 20bp sgRNA transcription module and 1kb donor DNA | This study |
| pHTDV | pHT01 derivative，containging *Cpf1* expression cassette，*ligD* targeting 23bp crRNA transcription module and 1.2kb donor DNA | This study |
| pHTsfGFPKi | pHT01 derivative，containging *Cpf1* expression cassette，*sacA* targeting 23bp crRNA transcription module and 1.2kb donor DNA for generating *sfGFP* insertion at *sacA* | This study |
| pHTCpf1 | pHT01 derivative plasmid，containing P_grac_-Cpf1 | This study |
| pAD123 | *E.coli*-*Bacillus* shuttle plasmid，promoter-less gfpmut3，*Cm*^r^ in *Bacillus* | Lab stock |
| pADsacA | pAD123 derivative，containing *sacA* targeting 23bp crRNA transcription module and 1.2kb donor DNA | This study |
| pBSG03 | *E.coli*-*B.subtilis* shuttle vector，P_srfA_ promoter，*gfp*，*Amp*^r^，*Kan*^r^ | Lab stock |
| pBsacA | Derived from pBSG03,P::P_veg_-sacAcrRNA，homologous arm of *sacA*，*Amp*^r^，*Kan*^r^ | This study |
| pBbac | Derived from pBSG03,P_srfA_::P_veg_-bacDcrRNA，homologous arm of *bacD*，*Amp*^r^，*Kan*^r^ | This study |
| pBpps | Derived from pBSG03,P_srfA_::P_veg_-ppsCcrRNA，homologous arm of *ppsC*，*Amp*^r^，*Kan*^r^ | This study |
| pBsfGFPKi | pBSG03 derivative，containging *sacA* targeting 23bp crRNA transcription module and 1.2kb donor DNA for generating *sfGFP* insertion at *sacA* | This study |
| pB-PvegW-SAKo | pBSG03 derivative，targeting *sacA* and *aprE* gene，*Amp*^r^，*Kan*^r^ | This study |
| pB-PvegM-SAKo | pBSG03 derivative，targeting *sacA* and *aprE* gene，*Amp*^r^，*Kan*^r^ | This study |
| Strains |  |  |
| *E.coli* JM109 | *recA*1, *supE*44 *endA*1 *hsdR*17 (^r−^k,m^+^k) *gyrA*96 *relA*1 thi (*lac*-*proAB*) F’[*traD*36 *proAB*^+^ *lacI*^q^  *lacZ*ΔM15] | Lab stock |
| *B. subtilis* 168 | *trpC*2 | Lab stock |
| *B.subtilis* comK | Derived from *B.subtilis* 168，*lacA*::P_xylA_-*comK*，*Erm*^r^ | Lab stock |
| *BS*-*ganA*'-*Cpf1* | Derived from *B.subtilis* 168，*lacA*::P_xylA_-*Cpf1*，Cm^r^ | This study |

**Supplementary Table 2. Primers used in this study**

| Primers | Sequences(5'-3') |
| --- | --- |
| pHT-P43-F | TACTGGTTTCATTTTTTTTCCTCCTTTTCTCGAGGGTA |
| pHT-P43-R | GTTAGCTTTTTCTGAAAATTCTTACATTTATTTTACATTTTTAGAAATG |
| pHT-P43-b-F | GTAAGAATTTTCAGAAAAAGCTAACGGAAAAGGGAG |
| pHT-P43-b-R | AGGAGGAAAAAAAATGAAACCAGTAACGTTATACGATG |
| P43-Fncpf1-F | CATTAATTGCGTTGCGCTTAGTTATTCCTATTCTGCACGAACT |
| P43-Fncpf1-R | GGAGGAAAAAAAATGTCAATTTATCAAGAATTTGTTAATAAATATAG |
| P43-Fncpf1-b-F | GATAAATTGACATTTTTTTTCCTCCTTTTCTCGAGGGTA |
| P43-Fncpf1-b-R | TAGGAATAACTAAGCGCAACGCAATTAATGTG |
| pHT-pVeg-sacAcrRNA-F | TCTTATCACTTATTAACGTTGATATAATTTAAATTTTATTTGACAAA |
| pHT-pVeg-sacAcrRNA-R | AGTTGCAGACAAAGGAGAGCGTTCACCGACAAA |
| pHT-sacAcr-b-F | TGAACGCTCTCCTTTGTCTGCAACTGAAAAGT |
| pHT-sacAcr-b-R | ATATCAACGTTAATAAGTGATAAGATAAAAAATTTTTCACGCTT |
| sacA-HA-F | CTGAAAAGTTTATACTACATAAGTGTCCAAATTCCGAC |
| sacA-HA-R | TGTTCCAGGTAAGGATGACAGCACATGACCAGG |
| pHT-HA-b-F | TGTGCTGTCATCCTTACCTGGAACAAATGGTTG |
| pHT-HA-b-R | CACTTATGTAGTATAAACTTTTCAGTTGCAGACAAAGGA |
| pHT-Pgrac-Cpf1-F | AAAGGAGGAAGGATCATGTCAATTTATCAAGAATTTGTTAATAAATATAGT |
| pHT-Pgrac-cpf1-R | CGTCGACTCTAGTTAGTTATTCCTATTCTGCACGAACT |
| pHT-Pgrac-Cpf1-b-F | TAGGAATAACTAACTAGAGTCGACGTCCCC |
| pHT-Pgrac-Cpf1-b-R | AAATTGACATGATCCTTCCTCCTTTAATTGGGA |
| pAD123-pVeg-sacAcrT-F | TAAGAAACCATTATTTTATTAACGTTGATATAATTTAAATTTTATTTGACAAAAATG |
| pAD123-pVeg-sacAcrT-R | CGAATTCTTGAAGACGGAGAGCGTTCACCGACAAA |
| pAD123-sacAcrT-b-F | CGGTGAACGCTCTCCGTCTTCAAGAATTCGAGCTC |
| pAD123-sacAcrT-b-R | TATCAACGTTAATAAAATAATGGTTTCTTAGACGTCAGGT |
| pAD-sacAH-F | GGCCCTTTCGTCTACATAAGTGTCCAAATTCCGAC |
| pAD-sacAH-R | TGAAATATGTTTTGATGACAGCACATGACCAGG |
| pAD-sacAH-b-F | TGTGCTGTCATCAAAACATATTTCAACACAATACAAATGG |
| pAD-sacAH-b-R | GGACACTTATGTAGACGAAAGGGCCTCGTGATA |
| lacA-Cpf1-F | GTGATGTCAAAGCTTGAAAAAAC |
| lacA-Cpf1-R | CTAATGTGTGTTTACGACAATTCT |
| pAX01-Cpf1-F | GGGGGAAATGGGATCCATGTCAATTTATCAAGAATTTGTTAATAAATATAG |
| pAX01-Cpf1-R | GCCTGCAGGTCGACTTAGTTATTCCTATTCTGCACGA |
| pAX01-Cpf1-b-F | GGAATAACTAAGTCGACCTGCAGGCA |
| pAX01-Cpf1-b-R | TGATAAATTGACATGGATCCCATTTCCCCC |
| pB-pVeg-sacAHA-F | GGGCAGGTTTATTAACGTTGATATAATTTAAATTTTATTTGACAAAAA |
| pB-pVeg-sacAHA-R | TCAGGCGAATTCATGACAGCACATGACCAGG |
| pB-pVeg-sacAHA-b-F | CATGTGCTGTCATGAATTCGCCTGATGCGGT |
| pB-pVeg-sacAHA-b-R | TATATCAACGTTAATAAACCTGCCCTCTGCCAC |
| pB-ppsCcrRENA-F | TAGATAGACAAAGTTCATCCAGCTCCAAATTTCAAATAAAACGAAAGGCTCAGTCGAAAG |
| pB-ppsCcrRNA-R | GGAGCTGGATGAACTTTGTCTATCTACAACAGTAGAAATTACATTTATTGTACAACACGA |
| pps-F | TGAAAAGTTTATACCCTTTTTTGGACGGCCAAT |
| pps-R | ATCAGGCGAATTCGCTCGAAGTGTTAAACAGTACG |
| pB-ppsCcrRNA-b-F | AACACTTCGAGCGAATTCGCCTGATGCGGTATTT |
| pB-ppsCcrRNA-b-R | TCCAAAAAAGGGTATAAACTTTTCAGTTGCAGACAAAGGAG |
| pB-sfGFP-KI-F | CGGCGCGTAAAAATGAGCAAAGGAGAAGAACTTTT |
| pB-sfGFP-KI-R | GAGTGGAGATTTTTATTTGTAGAGCTCATCCATGC |
| pB-sfGFP-KI-b-F | GCTCTACAAATAAAAATCTCCACTCTGTCAGGTT |
| pB-sfGFP-KI-b-R | CTCCTTTGCTCATTTTTACGCGCCGCAAAC |
| AmpM-BsaI-F | GTGAGCGTGGGTCCCGCGGTATCATTGCAGCAC |
| AmpM-BsaI-R | ATGATACCGCGGGACCCACGCTCACCGGCT |
| RepM-BsaI-F | GTGGAAAAGTGAGGCCATGGAGAGAAAAGAAAATCGCTA |
| RepM-BsaI-R | TCTCTCCATGGCCTCACTTTTCCACTTTTTGTCTTGT |
| pHT-ganAcr-F | ATCCTTGTTGACGTTATGCCAATTGATTTCAAATAAAACGAAAGGCTCAGTCGAAAG |
| pHT-ganAcr-R | CAATTGGCATAACGTCAACAAGGATCTACAACAGTAGAAATTACATTTATTGTACAACAC |
| PHT-all-ganAHA-F | CTGAAAAGTTTATAAGTAGTGAAAGTCGGCCAG |
| PHT-all-ganAHA-R | TTCCAGGTAAGGAGTTTTCAGCTTTAATTGCCTTGT |
| PHT-all-ganAHA-b-F | AAAGCTGAAAACTCCTTACCTGGAACAAATGGTTG |
| PHT-all-ganAHA-b-R | CTTTCACTACTTATAAACTTTTCAGTTGCAGACAAAGGA |
| pHT-DVcr-F | ATCGGTCCAAATACGAAAGAAGCGTATTTCAAATAAAACGAAAGGCTCAGTCGAAAG |
| pHT-DVcr-R | ACGCTTCTTTCGTATTTGGACCGATCTACAACAGTAGAAATTACATTTATTGTACAACAC |
| PHT-all-DVHA-F | GAAAAGTTTATACGTTAGGGTTTTATCGTCTCAT |
| PHT-all-DVHA-R | TCCAGGTAAGGTGGATTATAGATGAAGCATGCAAGC |
| PHT-all-DVHA-b-F | TCATCTATAATCCACCTTACCTGGAACAAATGGTTG |
| PHT-all-DVHA-b-R | TAAAACCCTAACGTATAAACTTTTCAGTTGCAGACAAAGG |
| sfGFPKI-F | CGGCGCGTAAAAATGAGCAAAGGAGAAGAACTTTT |
| sfGFPKI-R | GAGTGGAGATTTTTATTTGTAGAGCTCATCCATGC |
| pHT-all-sfGFPKI-b-F | GCTCTACAAATAAAAATCTCCACTCTGTCAGGTT |
| pHT-all-sfGFPKI-b-R | CTCCTTTGCTCATTTTTACGCGCCGCAAAC |
| pB-sacAHA-sfGFP-F | GGGCAGGTTTATTAACGTTGATATAATTTAAATTTTATTTGACAAAAA |
| pB-sacAHA-sfGFP-R | TCAGGCGAATTCATGACAGCACATGACCAGG |
| pB-sacAHA-sfGFP-b-F | CATGTGCTGTCATGAATTCGCCTGATGCGGT |
| pB-sacAHA-sfGFP-b-R | TATATCAACGTTAATAAACCTGCCCTCTGCCAC |
| pB-aprEHA-F | CAATCTGCTCTGAATTATGGGCCACGAAATGGG |
| pB-aprEHA-R | TAACTATGCGGCACCGATATTGGTTAAACAGCGG |
| pB-aprEHA-b-F | AACCAATATCGGTGCCGCATAGTTAAGCCAG |
| pB-aprEHA-b-R | GTGGCCCATAATTCAGAGCAGATTGTACTGAGAGT |
| pB-mCherry-F | TTCTTTTTACTAATGGTTTCTAAAGGCGAAGAAGATAA |
| pB-mCherry-R | GAGAGGGTAAAGATTATTTGTAAAGTTCATCCATGCCG |
| pB-mCherry-b-F | CTTTACAAATAATCTTTACCCTCTCCTTTTAAAAAAATTCAGA |
| pB-mCherry-b-R | CTTTAGAAACCATTAGTAAAAAGAAGCAGGTTCCTCCATA |
| BsaI-1-F | AATAAAAGATCCAAGAGACCTCTCTTGTATCTTTTTTATTTT |
| BsaI-1-R | AGAGAGGTCTCTTGGATCTTTTATTCAGCAATCGCGC |
| BsaI-2-F | AGCACGAGGTCTCAGCTTGAGCAAAACCCCCCTTTGC |
| BsaI-2-R | TTTGCTCAAGCTGAGACCTCGTGCTCGTTTAAAAATCAGCAAG |
| aprEcrRNA-BsaI-F | CGCGGGTCTCATCCATTATTAACGTTGATATAATTTAAATTTTATTTGACAAA |
| aprEcrRNA-BsaI-R | CGCTGGTCTCAAAGCGAGAGCGTTCACCGACAAA |
| SA-TB5-F | TGAATAAAAGATTATTAACGTTGATATAATTTAAATTTTATTTGACAAAAATGG |
| SA-TB5-R | GTTGCAGACAAAGGCTTTGACATCATAGCCCATCTC |
| SA-TB5-b-F | TGATGTCAAAGCCTTTGTCTGCAACTGAAAAGTTTATACTAC |
| SA-TB5-b-R | ATCAACGTTAATAATCTTTTATTCAGCAATCGCGC |
| Bac-crRNA-F | AAATGCTGCGGGTACAAATGGCAATTTCAAATAAAACGAAAGGCTCAGTCGAAAGA |
| Bac-crRNA-R | TGCCATTTGTACCCGCAGCATTTATCTACAACAGTAGAAATTACATTTATTGTAC |
| pHT-Cas9-AIO-F | ATTGCGTTGCGCTCAGTCACCTCCTAGCTGAC |
| pHT-Cas9-AIO-R | GGAGGAAAAAAAATGGATAAGAAATACTCAATAGGCTTAG |
| pHT-Cas9-AIO-b-F | ATTTCTTATCCATTTTTTTTCCTCCTTTTCTCGAGGG |
| pHT-Cas9-AIO-b-R | AGGAGGTGACTGAGCGCAACGCAATTAATGTG |
| pHT-lacAsg-AIO-F | TCTTATCACTTATTAACGTTGATATAATTTAAATTTTATTTGACAAAAATG |
| pHT-lacAsg-AIO-R | GTTGCAGACAAAGAAAAAAGCACCGACTCGGT |
| pHT-lacAsg-AIO-b-F | CGGTGCTTTTTTCTTTGTCTGCAACTGAAAAGT |
| pHT-lacAsg-AIO-b-R | ATATCAACGTTAATAAGTGATAAGATAAAAAATTTTTCACGCTT |

**Supplementary Table 3. crRNA used in this study**

| crRNA Sequences(5'-3') | PAM | Purpose |
| --- | --- | --- |
| TCGGCCGCCTCGATTATAACAAG | TTTG | Deleted *sacA* |
| CCTTGTTGACGTTATGCCAATTG | TTTG | Deleted *ganA* |
| CGGTCCAAATACGAAAGAAGCGT | TTTG | Deleted *ligV* and *ligD* |
| TCGGCCGCCTCGATTATAACAAG | TTTG | Inserted *sfGFP* into *sacA* locus |
| AAATGCTGCGGGTACAAATGGCA | TTTG | Deleted *bac* operon |
| AGACAAAGTTCATCCAGCTCCAA | TTTG | Deleted *pps* operon |
| TTTCAGAAGGTACGAAGCTTGCT | TTTG | Deleted *aprE* |

**Supplementary Table 4. sgRNA used in this study**

| sgRNA Sequences(5'-3') | PAM | Purpose |
| --- | --- | --- |
| ATGGAGCGCACTTGAGCCGG | AGG | Deleted *ganA* |

**Supplementary Table 5. The total number of transformants of different construction type**

| Type of contruction | Total number of transformants |
| --- | --- |
| AIO | 484 |
| TPs | 306 |
| CCB-CIGE | 544 |

**Supplementary Figure**


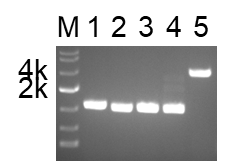


**Supplementary Figure 1.** Verification of the existence of *pps* operon in *Bacillus subtilis* before knocking out *pps* operon by CCB-CIGE system. *pps* operon was composed of five genes: *ppsA*, *ppsB*, *ppsC*, *ppsD* and *ppsE*. Schematic diagram of colony PCR for wild-type *Bacillus subtilis* 168 with customized primers. Lane “M” in the figure represents DNA marker. The lanes 1 to 5 is the cPCR result of *ppsA*, *ppsB*, *ppsC*, *ppsD* and *ppsE*, respectively.


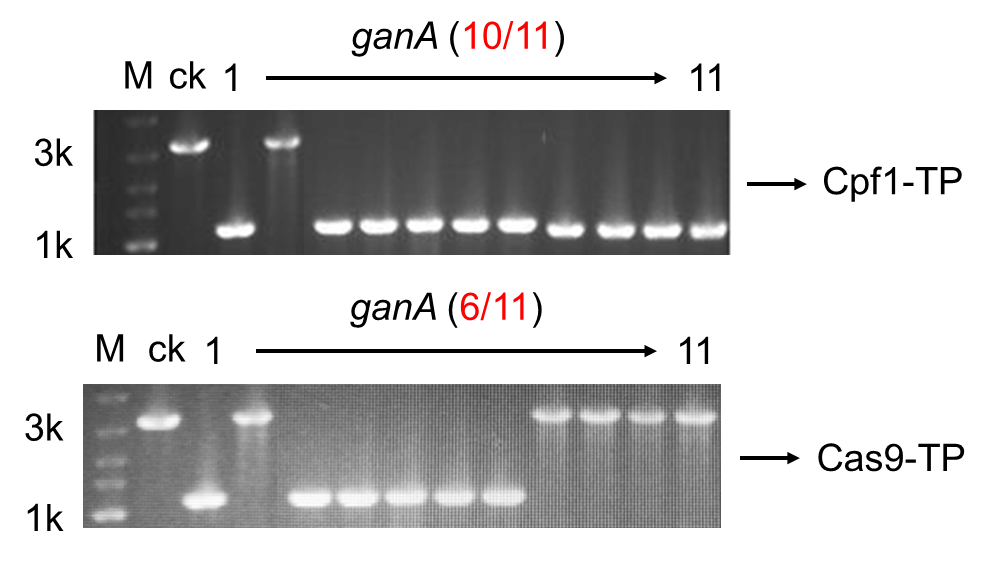


**Supplementary Figure 2.** Comparison of the efficiency of CRISPR-Cpf1 and CRISPR-Cas9 systems (Two plasmids system) in knockout of *ganA* gene in *Bacillus subtilis* 168. M and ck represent the 5-kb DNA ladder and wild-type *B. subtilis* 168, respectively. Lane1-11, PCR amplificaiton with the mutant gDNA using external primer.

**Supplementary Protocol**

**How to use AIO system**

First of all, to construct the AIO platform, we need to express Cpf1 and corresponding crRNA on one plasmid at the same time. Here, we choose pHT01 as the skeleton of AIO. Through the proper modification of the pHT01 vector, we have obtained the AIO platform based on CRISPR-Cpf1. In this platform, Cpf1 and crRNA are constitutively expressed by P43 and P_veg_ promoters, respectively (detailed construction process refer to materials and methods). Next, we need to transform this AIO-plasmid into *Bacillus subtilis* 168 (transformaiton procedure refer to Anagnostopoulos and Spizizen, 1961). The transformants were randomly picked up and inoculated into 5 mL LB medium for further overnight culture (culture conditions refer to results and discussion). The next day, the culture was spread onto the LB plate with the corresponding antibiotic. Then, we can select the transformants for colony PCR (cPCR) and Sanger sequencing to obtain our desired mutants.

**How to use TP system**

Construction of two-plasmids (TPs) system required to construct two plasmids to express FnCpf1 nuclease and corresponding crRNA, respectively. Here, we selected pHT01 and pAD123 as the backbone of harboring FnCpf1 nuclease and crRNA expression, respectively. In this platform, FnCpf1 nuclease was under the control of P_grac_ promoter and crRNA was constitutively expressed by P_veg_ promoter. Accordingly, the two plasmids were transformed into *B. subtilis* 168 in sequence (transformation procedure refer to Anagnostopoulos and Spizizen, 1961). Here, the plasmid pHT01-Cpf1containing FnCpf1 nuclease was firstly transformed into *B. subtilis* 168. Then, another plasmid (this paper takes pADsacA as an example) was transformed into the former transformant. Finally, we obtained the expected *B. subtilis* 168 with two plasmids. Then, the transformants were picked up, and then inoculated into 5mL-LB medium for propagation. When OD_600_ reached approximately 0.5, Isopropyl-*β*-D-thiogalactopyranoside (IPTG) with final concentration of 1 mM was added into the culture to induce the expression of FnCpf1. After 12-hour induction, the culture was spread onto LB agar plates. Then, incubated overnight prior to picking up transformants for colony PCR verification.

**How to use CCB-CIGE system**

When using this system, we first need to construct a recombinant *Bacillus subtilis* 168, whose chromosome integrates Cpf1 expression cassette. Here, we use xylose-induced promoter to drive Cpf1 expression, and integrate the whole expression cassette into the *ganA* site of *B. subtilis* 168. Since there is additional chloramphenicol resistance screening marker on the whole expression cassette, it is convenient for us to screen positive transformants. Finally, we produced the recombinant *B. subtilis* 168 (BS-*ganA'*-Cpf1, detailed construction process refer to materials and methods). Next, we need to construct a crRNA expression plasmid. Here, we selected high copy vector pBSG as the expression plasmid of crRNA. Like the AIO strategy, we also choose P_veg_ promoter to constitutively express crRNA. We can easily replace the corresponding crRNA by reverse PCR. When the recombinant *B. subtilis* 168 (BS-*ganA'*-Cpf1) and crRNA expression plasmids are constructed, we can use CCB-CIGE to modify any target gene accordingly. First of all, we need to introduce the crRNA targeting plasmid into the recombinant *B. subtilis* 168. The transformants were randomly picked and inoculated into 5-mL LB medium with a final concentration of 1% xylose and induced for about 12-16 hours. The culture was then gradient diluted and spread onto the LB plate containing the corresponding antibiotics. When a single colony can be clearly observed on the plate, we can pick and perform cPCR and sanger sequencing verification.

**Supplementary method**

**Construction of all-in-one system (****pHTganA-Cpf1 and pHTganA-Cas9)**

To produce plasmids for the deletion of *ganA* gene based on CRISPR-Cpf1 and CRISPR-Cas9, we first used pHTsacA plasmid as a template to carry out reverse PCR by using the primers pHT-ganAcr-F/R to obtain the crRNA targeting *ganA*. Finally, we obtained the pHT-ganAcrRNA-sacAHA plasmid. Previously, we have successfully constructed pB-ganAHA, which contained the 1000 bp homologous arms of *ganA*. Here, we amplified the homologous arms of *ganA* from the pB-ganAHA using the primers pHT-all-ganAHA-F/R, while the backbone of the plasmid, harboring Cpf1 and ganAcrRNA, was amplified using the primers pHT-all-ganAHA-b-F/R. Then, the homologous arms of *ganA* was inserted into the shuttle vector pHT-ganAcrRNA-sacAHA using the Gibson assembly, yielding pHTganA-Cpf1. For construction of pHTganA-Cas9 plasmid, we performed the following operations with pHTganA-Cpf1 as the template. First, the *cas9* gene was amplified by primers pHT-Cas9-AIO-F/R using pCas9 (addgene #42876) as template, and then the skeleton was amplified by primers pHT-Cas9-AIO-b-F/R using pHTganA-Cpf1 as template. Finally, *cas9* gene fragment and skeleton were stitched by Gibson assembly (Cpf1 replaced by Cas9), producing pHT-Cas9. Then, the sgRNA expression cassette targeting *ganA* was amplified by primers pHT-lacAsg-AIO-F/R using pADlacA (containing the sgRNA expression cassette targeting *ganA*) as a template. The skeleton of sgRNA was amplified by primers pHT-lacAsg-AIO-b-F/R using pHT-Cas9 as template. Finally, the two fragments are connected by Gibson assembly, yielding pHTganA-Cas9.

**Construction of all-in-one system (pHTDV)**

To construct pHTDV to delete the *ligD* ＆ *ligV*, oligonucleotides of 23-bp crRNA (pHT-DVcr-F and pHT-DVcr-R) were synthesized and then cloned into pHTsacA by reverse PCR, producing pHT-DVcrRNA-sacAHA. Furthermore, the homologous arms of *ligD* ＆ *ligV* (each was 600 bp in length) were amplified from pB-DVHA using the primers pHT-all-DVHA-F/R. At the same time, the backbone of the plasmid harboring *Cpf1* and DVcrRNA sequence was amplified using the primers pHT-all-DVHA-b-F/R. Meanwhile, the homologous arms of *ligD* ＆ *ligV* were inserted into the plasmid pHT-DVcrRNA-sacAHA using the Gibson assembly, yielding pHTDV.

**Construction of chromosomally integrated genome editing system (CIGE, pBbac and pBpps)**

To delete *bac* operon, we first employed the plasmid pBsacA as a template to obtain the plasmid pB-baccrRNA-sacAHA with crRNA targeting *bacD* by reverse PCR using primers Bac-crRNA-F and Bac-crRNA-R. To generate a donor DNA template for deletion of *bac* operon, the upstream and downstream homologous arms (each was of 500 bp in length) of *bac* operon were amplified using primer pairs Bac-up-F/R and Bac-down-F/R, respectively. Fusion PCR using primers pB-bacKo-F/R was performed to generate a 1-kb donor DNA template for bac operon deletion, termed pBbac.

Similarly, to generate the deletion mutant of *pps* operon, the 23-bp crRNAs (pB-ppsCcrRNA-F and pB-ppsCcrRNA-R) were synthesized using pBsacA as template for reverse PCR, producing pB-ppsCcrRNA-sacAHA. To generate a 1.6-kb donor DNA template (each was of 800 bp in length) used to deletion of *pps* operon, we used primers pps-F/R and pB-ppsCcrRNA-b-F/R to amplify the donor DNA template and plasmid backbone (pB-ppsCcrRNA-sacAHA), respectively. Then, the two PCR products were assembled by Gibson Assembly as aforementioned, yielding pBpps.

**Supplementary Sequences**

***sacA*-targeting crRNA Sequences**

**TTATTAACGTTGATATAATTTAAATTTTATTTGACAAAAATGGGCTCGTGTTGTACAATAAATGTAATTTCTACTGTTGTAGATTCGGCCGCCTCGATTATAACAAGATTTCAAATAAAACGAAAGGCTCAGTCGAAAGACTGGGCCTTTCGTTTTATCTGTTGTTTGTCGGTGAACGCTCTCCTTTGTCTGCAACTGAAAAGTTTATA**

***ganA*-targeting crRNA Sequences**

**TTATTAACGTTGATATAATTTAAATTTTATTTGACAAAAATGGGCTCGTGTTGTACAATAAATGTAATTTCTACTGTTGTAGATCCTTGTTGACGTTATGCCAATTGATTTCAAATAAAACGAAAGGCTCAGTCGAAAGACTGGGCCTTTCGTTTTATCTGTTGTTTGTCGGTGAACGCTCTCCTTTGTCTGCAACTGAAAAGTTTATA**

***ligV and ligD*-targeting crRNA Sequences**

**TTATTAACGTTGATATAATTTAAATTTTATTTGACAAAAATGGGCTCGTGTTGTACAATAAATGTAATTTCTACTGTTGTAGATCGGTCCAAATACGAAAGAAGCGTATTTCAAATAAAACGAAAGGCTCAGTCGAAAGACTGGGCCTTTCGTTTTATCTGTTGTTTGTCGGTGAACGCTCTCCTTTGTCTGCAACTGAAAAGTTTATA**

***aprE*-targeting crRNA Sequences**

**TTATTAACGTTGATATAATTTAAATTTTATTTGACAAAAATGGGCTCGTGTTGTACAATAAATGTAATTTCTACTGTTGTAGATTTTCAGAAGGTACGAAGCTTGCTATTTCAAATAAAACGAAAGGCTCAGTCGAAAGACTGGGCCTTTCGTTTTATCTGTTGTTTGTCGGTGAACGCTCTCCTTTGTCTGCAACTGAAAAGTTTATA**

***bacD*-targeting crRNA Sequences**

**TTATTAACGTTGATATAATTTAAATTTTATTTGACAAAAATGGGCTCGTGTTGTACAATAAATGTAATTTCTACTGTTGTAGATAAATGCTGCGGGTACAAATGGCAATTTCAAATAAAACGAAAGGCTCAGTCGAAAGACTGGGCCTTTCGTTTTATCTGTTGTTTGTCGGTGAACGCTCTCCTTTGTCTGCAACTGAAAAGTTTATA**

***ppsC*-targeting crRNA Sequences**

**TTATTAACGTTGATATAATTTAAATTTTATTTGACAAAAATGGGCTCGTGTTGTACAATAAATGTAATTTCTACTGTTGTAGATAGACAAAGTTCATCCAGCTCCAAATTTCAAATAAAACGAAAGGCTCAGTCGAAAGACTGGGCCTTTCGTTTTATCTGTTGTTTGTCGGTGAACGCTCTCCTTTGTCTGCAACTGAAAAGTTTATA**

***sacA*- and *aprE*-targeting crRNA Sequences**

**TTATTAACGTTGATATAATTTAAATTTTATTTGACAAAAATGGGCTCGTGTTGTGCAATAAATGTAATTTCTACTGTTGTAGATTTTCAGAAGGTACGAAGCTTGCTAATTTCTACTGTTGTAGATTCGGCCGCCTCGATTATAACAAGAATTTCTACTGTTGTAGATATTTCAGGACACCGTTCAAATTGAACGGTGTTTTTCTTTGAAAAGAGAGATGGGCTATGATGTCAAAGCCTTTGTCTGCAACTGAAAAGTTTATA**

**Annotations:**

**Wild type P_veg_ promoter, Direct repeat sequence, 23 base target site, rrnB T1 terminator, Mutant of P_veg_ promoter, TB5 terminator**

***ganA*-targeting sgRNA Sequence**

**TTATTAACGTTGATATAATTTAAATTTTATTTGACAAAAATGGGCTCGTGTTGTACAATAAATGTATGGAGCGCACTTGAGCCGGGTTTTAGAGCTAGAAATAGCAAGTTAAAATAAGGCTAGTCCGTTATCAACTTGAAAAAGTGGCACCGAGTCGGTGCTTTTTT**

**Annotations:**

**Wild type P_veg_ promoter, 23 base target site, tracr RNA terminator**

***Francisella novicida* *U112* Cpf1 (*Fn*Cpf1) nucleotide sequence (refer to addgene #69973, pY001 Plasmid)**

**ATGTCAATTTATCAAGAATTTGTTAATAAATATAGTTTAAGTAAAACTCTAAGATTTGAGTTAATCCCACAGGGTAAAACACTTGAAAACATAAAAGCAAGAGGTTTGATTTTAGATGATGAGAAAAGAGCTAAAGACTACAAAAAGGCTAAACAAATAATTGATAAATATCATCAGTTTTTTATAGAGGAGATATTAAGTTCGGTTTGTATTAGCGAAGATTTATTACAAAACTATTCTGATGTTTATTTTAAACTTAAAAAGAGTGATGATGATAATCTACAAAAAGATTTTAAAAGTGCAAAAGATACGATAAAGAAACAAATATCTGAATATATAAAGGACTCAGAGAAATTTAAGAATTTGTTTAATCAAAACCTTATCGATGCTAAAAAAGGGCAAGAGTCAGATTTAATTCTATGGCTAAAGCAATCTAAGGATAATGGTATAGAACTATTTAAAGCCAATAGTGATATCACAGATATAGATGAGGCGTTAGAAATAATCAAATCTTTTAAAGGTTGGACAACTTATTTTAAGGGTTTTCATGAAAATAGAAAAAATGTTTATAGTAGCAATGATATTCCTACATCTATTATTTATAGGATAGTAGATGATAATTTGCCTAAATTTCTAGAAAATAAAGCTAAGTATGAGAGTTTAAAAGACAAAGCTCCAGAAGCTATAAACTATGAACAAATTAAAAAAGATTTGGCAGAAGAGCTAACCTTTGATATTGACTACAAAACATCTGAAGTTAATCAAAGAGTTTTTTCACTTGATGAAGTTTTTGAGATAGCAAACTTTAATAATTATCTAAATCAAAGTGGTATTACTAAATTTAATACTATTATTGGTGGTAAATTTGTAAATGGTGAAAATACAAAGAGAAAAGGTATAAATGAATATATAAATCTATACTCACAGCAAATAAATGATAAAACACTCAAAAAATATAAAATGAGTGTTTTATTTAAGCAAATTTTAAGTGATACAGAATCTAAATCTTTTGTAATTGATAAGTTAGAAGATGATAGTGATGTAGTTACAACGATGCAAAGTTTTTATGAGCAAATAGCAGCTTTTAAAACAGTAGAAGAAAAATCTATTAAAGAAACACTATCTTTATTATTTGATGATTTAAAAGCTCAAAAACTTGATTTGAGTAAAATTTATTTTAAAAATGATAAATCTCTTACTGATCTATCACAACAAGTTTTTGATGATTATAGTGTTATTGGTACAGCGGTACTAGAATATATAACTCAACAAATAGCACCTAAAAATCTTGATAACCCTAGTAAGAAAGAGCAAGAATTAATAGCCAAAAAAACTGAAAAAGCAAAATACTTATCTCTAGAAACTATAAAGCTTGCCTTAGAAGAATTTAATAAGCATAGAGATATAGATAAACAGTGTAGGTTTGAAGAAATACTTGCAAACTTTGCGGCTATTCCGATGATATTTGATGAAATAGCTCAAAACAAAGACAATTTGGCACAGATATCTATCAAATATCAAAATCAAGGTAAAAAAGACCTACTTCAAGCTAGTGCGGAAGATGATGTTAAAGCTATCAAGGATCTTTTAGATCAAACTAATAATCTCTTACATAAACTAAAAATATTTCATATTAGTCAGTCAGAAGATAAGGCAAATATTTTAGACAAGGATGAGCATTTTTATCTAGTATTTGAGGAGTGCTACTTTGAGCTAGCGAATATAGTGCCTCTTTATAACAAAATTAGAAACTATATAACTCAAAAGCCATATAGTGATGAGAAATTTAAGCTCAATTTTGAGAACTCGACTTTGGCTAATGGTTGGGATAAAAATAAAGAGCCTGACAATACGGCAATTTTATTTATCAAAGATGATAAATATTATCTGGGTGTGATGAATAAGAAAAATAACAAAATATTTGATGATAAAGCTATCAAAGAAAATAAAGGCGAGGGTTATAAAAAAATTGTTTATAAACTTTTACCTGGCGCAAATAAAATGTTACCTAAGGTTTTCTTTTCTGCTAAATCTATAAAATTTTATAATCCTAGTGAAGATATACTTAGAATAAGAAATCATTCCACACATACAAAAAATGGTAGTCCTCAAAAAGGATATGAAAAATTTGAGTTTAATATTGAAGATTGCCGAAAATTTATAGATTTTTATAAACAGTCTATAAGTAAGCATCCGGAGTGGAAAGATTTTGGATTTAGATTTTCTGATACTCAAAGATATAATTCTATAGATGAATTTTATAGAGAAGTTGAAAATCAAGGCTACAAACTAACTTTTGAAAATATATCAGAGAGCTATATTGATAGCGTAGTTAATCAGGGTAAATTGTACCTATTCCAAATCTATAATAAAGATTTTTCAGCTTATAGCAAAGGGCGACCAAATCTACATACTTTATATTGGAAAGCGCTGTTTGATGAGAGAAATCTTCAAGATGTGGTTTATAAGCTAAATGGTGAGGCAGAGCTTTTTTATCGTAAACAATCAATACCTAAAAAAATCACTCACCCAGCTAAAGAGGCAATAGCTAATAAAAACAAAGATAATCCTAAAAAAGAGAGTGTTTTTGAATATGATTTAATCAAAGATAAACGCTTTACTGAAGATAAGTTTTTCTTTCACTGTCCTATTACAATCAATTTTAAATCTAGTGGAGCTAATAAGTTTAATGATGAAATCAATTTATTGCTAAAAGAAAAAGCAAATGATGTTCATATATTAAGTATAGATAGAGGTGAAAGACATTTAGCTTACTATACTTTGGTAGATGGTAAAGGCAATATCATCAAACAAGATACTTTCAACATCATTGGTAATGATAGAATGAAAACAAACTACCATGATAAGCTTGCTGCAATAGAGAAAGATAGGGATTCAGCTAGGAAAGACTGGAAAAAGATAAATAACATCAAAGAGATGAAAGAGGGCTATCTATCTCAGGTAGTTCATGAAATAGCTAAGCTAGTTATAGAGTATAATGCTATTGTGGTTTTTGAGGATTTAAATTTTGGATTTAAAAGAGGGCGTTTCAAGGTAGAGAAGCAGGTCTATCAAAAGTTAGAAAAAATGCTAATTGAGAAACTAAACTATCTAGTTTTCAAAGATAATGAGTTTGATAAAACTGGGGGAGTGCTTAGAGCTTATCAGCTAACAGCACCTTTTGAGACTTTTAAAAAGATGGGTAAACAAACAGGTATTATCTACTATGTACCAGCTGGTTTTACTTCAAAAATTTGTCCTGTAACTGGTTTTGTAAATCAGTTATATCCTAAGTATGAAAGTGTCAGCAAATCTCAAGAGTTCTTTAGTAAGTTTGACAAGATTTGTTATAACCTTGATAAGGGCTATTTTGAGTTTAGTTTTGATTATAAAAACTTTGGTGACAAGGCTGCCAAAGGCAAGTGGACTATAGCTAGCTTTGGGAGTAGATTGATTAACTTTAGAAATTCAGATAAAAATCATAATTGGGATACTCGAGAAGTTTATCCAACTAAAGAGTTGGAGAAATTGCTAAAAGATTATTCTATCGAATATGGGCATGGCGAATGTATCAAAGCAGCTATTTGCGGTGAGAGCGACAAAAAGTTTTTTGCTAAGCTAACTAGTGTCCTAAATACTATCTTACAAATGCGTAACTCAAAAACAGGTACTGAGTTAGATTATCTAATTTCACCAGTAGCAGATGTAAATGGCAATTTCTTTGATTCGCGACAGGCGCCAAAAAATATGCCTCAAGATGCTGATGCCAATGGTGCTTATCATATTGGGCTAAAAGGTCTGATGCTACTAGGTAGGATCAAAAATAATCAAGAGGGCAAAAAACTCAATTTGGTTATCAAAAATGAAGAGTATTTTGAGTTCGTGCAGAATAGGAATAACTAA**

***Francisella novicida* *U112* Cpf1 (*Fn*Cpf1) amino acid sequence**

**MSIYQEFVNKYSLSKTLRFELIPQGKTLENIKARGLILDDEKRAKDYKKAKQIIDKYHQFFIEEILSSVCISEDLLQNYSDVYFKLKKSDDDNLQKDFKSAKDTIKKQISEYIKDSEKFKNLFNQNLIDAKKGQESDLILWLKQSKDNGIELFKANSDITDIDEALEIIKSFKGWTTYFKGFHENRKNVYSSNDIPTSIIYRIVDDNLPKFLENKAKYESLKDKAPEAINYEQIKKDLAEELTFDIDYKTSEVNQRVFSLDEVFEIANFNNYLNQSGITKFNTIIGGKFVNGENTKRKGINEYINLYSQQINDKTLKKYKMSVLFKQILSDTESKSFVIDKLEDDSDVVTTMQSFYEQIAAFKTVEEKSIKETLSLLFDDLKAQKLDLSKIYFKNDKSLTDLSQQVFDDYSVIGTAVLEYITQQIAPKNLDNPSKKEQELIAKKTEKAKYLSLETIKLALEEFNKHRDIDKQCRFEEILANFAAIPMIFDEIAQNKDNLAQISIKYQNQGKKDLLQASAEDDVKAIKDLLDQTNNLLHKLKIFHISQSEDKANILDKDEHFYLVFEECYFELANIVPLYNKIRNYITQKPYSDEKFKLNFENSTLANGWDKNKEPDNTAILFIKDDKYYLGVMNKKNNKIFDDKAIKENKGEGYKKIVYKLLPGANKMLPKVFFSAKSIKFYNPSEDILRIRNHSTHTKNGSPQKGYEKFEFNIEDCRKFIDFYKQSISKHPEWKDFGFRFSDTQRYNSIDEFYREVENQGYKLTFENISESYIDSVVNQGKLYLFQIYNKDFSAYSKGRPNLHTLYWKALFDERNLQDVVYKLNGEAELFYRKQSIPKKITHPAKEAIANKNKDNPKKESVFEYDLIKDKRFTEDKFFFHCPITINFKSSGANKFNDEINLLLKEKANDVHILSIDRGERHLAYYTLVDGKGNIIKQDTFNIIGNDRMKTNYHDKLAAIEKDRDSARKDWKKINNIKEMKEGYLSQVVHEIAKLVIEYNAIVVFEDLNFGFKRGRFKVEKQVYQKLEKMLIEKLNYLVFKDNEFDKTGGVLRAYQLTAPFETFKKMGKQTGIIYYVPAGFTSKICPVTGFVNQLYPKYESVSKSQEFFSKFDKICYNLDKGYFEFSFDYKNFGDKAAKGKWTIASFGSRLINFRNSDKNHNWDTREVYPTKELEKLLKDYSIEYGHGECIKAAICGESDKKFFAKLTSVLNTILQMRNSKTGTELDYLISPVADVNGNFFDSRQAPKNMPQDADANGAYHIGLKGLMLLGRIKNNQEGKKLNLVIKNEEYFEFVQNRNN***

***Streptococcus pyogenes* Cas9 (*Sp*Cas9) nucleotide sequence (refer to addgene #42876, pCas9 Plasmid)**

**ATGGATAAGAAATACTCAATAGGCTTAGATATCGGCACAAATAGCGTCGGATGGGCGGTGATCACTGATGAATATAAGGTTCCGTCTAAAAAGTTCAAGGTTCTGGGAAATACAGACCGCCACAGTATCAAAAAAAATCTTATAGGGGCTCTTTTATTTGACAGTGGAGAGACAGCGGAAGCGACTCGTCTCAAACGGACAGCTCGTAGAAGGTATACACGTCGGAAGAATCGTATTTGTTATCTACAGGAGATTTTTTCAAATGAGATGGCGAAAGTAGATGATAGTTTCTTTCATCGACTTGAAGAGTCTTTTTTGGTGGAAGAAGACAAGAAGCATGAACGTCATCCTATTTTTGGAAATATAGTAGATGAAGTTGCTTATCATGAGAAATATCCAACTATCTATCATCTGCGAAAAAAATTGGTAGATTCTACTGATAAAGCGGATTTGCGCTTAATCTATTTGGCCTTAGCGCATATGATTAAGTTTCGTGGTCATTTTTTGATTGAGGGAGATTTAAATCCTGATAATAGTGATGTGGACAAACTATTTATCCAGTTGGTACAAACCTACAATCAATTATTTGAAGAAAACCCTATTAACGCAAGTGGAGTAGATGCTAAAGCGATTCTTTCTGCACGATTGAGTAAATCAAGACGATTAGAAAATCTCATTGCTCAGCTCCCCGGTGAGAAGAAAAATGGCTTATTTGGGAATCTCATTGCTTTGTCATTGGGTTTGACCCCTAATTTTAAATCAAATTTTGATTTGGCAGAAGATGCTAAATTACAGCTTTCAAAAGATACTTACGATGATGATTTAGATAATTTATTGGCGCAAATTGGAGATCAATATGCTGATTTGTTTTTGGCAGCTAAGAATTTATCAGATGCTATTTTACTTTCAGATATCCTAAGAGTAAATACTGAAATAACTAAGGCTCCCCTATCAGCTTCAATGATTAAACGCTACGATGAACATCATCAAGACTTGACTCTTTTAAAAGCTTTAGTTCGACAACAACTTCCAGAAAAGTATAAAGAAATCTTTTTTGATCAATCAAAAAACGGATATGCAGGTTATATTGATGGGGGAGCTAGCCAAGAAGAATTTTATAAATTTATCAAACCAATTTTAGAAAAAATGGATGGTACTGAGGAATTATTGGTGAAACTAAATCGTGAAGATTTGCTGCGCAAGCAACGGACCTTTGACAACGGCTCTATTCCCCATCAAATTCACTTGGGTGAGCTGCATGCTATTTTGAGAAGACAAGAAGACTTTTATCCATTTTTAAAAGACAATCGTGAGAAGATTGAAAAAATCTTGACTTTTCGAATTCCTTATTATGTTGGTCCATTGGCGCGTGGCAATAGTCGTTTTGCATGGATGACTCGGAAGTCTGAAGAAACAATTACCCCATGGAATTTTGAAGAAGTTGTCGATAAAGGTGCTTCAGCTCAATCATTTATTGAACGCATGACAAACTTTGATAAAAATCTTCCAAATGAAAAAGTACTACCAAAACATAGTTTGCTTTATGAGTATTTTACGGTTTATAACGAATTGACAAAGGTCAAATATGTTACTGAAGGAATGCGAAAACCAGCATTTCTTTCAGGTGAACAGAAGAAAGCCATTGTTGATTTACTCTTCAAAACAAATCGAAAAGTAACCGTTAAGCAATTAAAAGAAGATTATTTCAAAAAAATAGAATGTTTTGATAGTGTTGAAATTTCAGGAGTTGAAGATAGATTTAATGCTTCATTAGGTACCTACCATGATTTGCTAAAAATTATTAAAGATAAAGATTTTTTGGATAATGAAGAAAATGAAGATATCTTAGAGGATATTGTTTTAACATTGACCTTATTTGAAGATAGGGAGATGATTGAGGAAAGACTTAAAACATATGCTCACCTCTTTGATGATAAGGTGATGAAACAGCTTAAACGTCGCCGTTATACTGGTTGGGGACGTTTGTCTCGAAAATTGATTAATGGTATTAGGGATAAGCAATCTGGCAAAACAATATTAGATTTTTTGAAATCAGATGGTTTTGCCAATCGCAATTTTATGCAGCTGATCCATGATGATAGTTTGACATTTAAAGAAGACATTCAAAAAGCACAAGTGTCTGGACAAGGCGATAGTTTACATGAACATATTGCAAATTTAGCTGGTAGCCCTGCTATTAAAAAAGGTATTTTACAGACTGTAAAAGTTGTTGATGAATTGGTCAAAGTAATGGGGCGGCATAAGCCAGAAAATATCGTTATTGAAATGGCACGTGAAAATCAGACAACTCAAAAGGGCCAGAAAAATTCGCGAGAGCGTATGAAACGAATCGAAGAAGGTATCAAAGAATTAGGAAGTCAGATTCTTAAAGAGCATCCTGTTGAAAATACTCAATTGCAAAATGAAAAGCTCTATCTCTATTATCTCCAAAATGGAAGAGACATGTATGTGGACCAAGAATTAGATATTAATCGTTTAAGTGATTATGATGTCGATCACATTGTTCCACAAAGTTTCCTTAAAGACGATTCAATAGACAATAAGGTCTTAACGCGTTCTGATAAAAATCGTGGTAAATCGGATAACGTTCCAAGTGAAGAAGTAGTCAAAAAGATGAAAAACTATTGGAGACAACTTCTAAACGCCAAGTTAATCACTCAACGTAAGTTTGATAATTTAACGAAAGCTGAACGTGGAGGTTTGAGTGAACTTGATAAAGCTGGTTTTATCAAACGCCAATTGGTTGAAACTCGCCAAATCACTAAGCATGTGGCACAAATTTTGGATAGTCGCATGAATACTAAATACGATGAAAATGATAAACTTATTCGAGAGGTTAAAGTGATTACCTTAAAATCTAAATTAGTTTCTGACTTCCGAAAAGATTTCCAATTCTATAAAGTACGTGAGATTAACAATTACCATCATGCCCATGATGCGTATCTAAATGCCGTCGTTGGAACTGCTTTGATTAAGAAATATCCAAAACTTGAATCGGAGTTTGTCTATGGTGATTATAAAGTTTATGATGTTCGTAAAATGATTGCTAAGTCTGAGCAAGAAATAGGCAAAGCAACCGCAAAATATTTCTTTTACTCTAATATCATGAACTTCTTCAAAACAGAAATTACACTTGCAAATGGAGAGATTCGCAAACGCCCTCTAATCGAAACTAATGGGGAAACTGGAGAAATTGTCTGGGATAAAGGGCGAGATTTTGCCACAGTGCGCAAAGTATTGTCCATGCCCCAAGTCAATATTGTCAAGAAAACAGAAGTACAGACAGGCGGATTCTCCAAGGAGTCAATTTTACCAAAAAGAAATTCGGACAAGCTTATTGCTCGTAAAAAAGACTGGGATCCAAAAAAATATGGTGGTTTTGATAGTCCAACGGTAGCTTATTCAGTCCTAGTGGTTGCTAAGGTGGAAAAAGGGAAATCGAAGAAGTTAAAATCCGTTAAAGAGTTACTAGGGATCACAATTATGGAAAGAAGTTCCTTTGAAAAAAATCCGATTGACTTTTTAGAAGCTAAAGGATATAAGGAAGTTAAAAAAGACTTAATCATTAAACTACCTAAATATAGTCTTTTTGAGTTAGAAAACGGTCGTAAACGGATGCTGGCTAGTGCCGGAGAATTACAAAAAGGAAATGAGCTGGCTCTGCCAAGCAAATATGTGAATTTTTTATATTTAGCTAGTCATTATGAAAAGTTGAAGGGTAGTCCAGAAGATAACGAACAAAAACAATTGTTTGTGGAGCAGCATAAGCATTATTTAGATGAGATTATTGAGCAAATCAGTGAATTTTCTAAGCGTGTTATTTTAGCAGATGCCAATTTAGATAAAGTTCTTAGTGCATATAACAAACATAGAGACAAACCAATACGTGAACAAGCAGAAAATATTATTCATTTATTTACGTTGACGAATCTTGGAGCTCCCGCTGCTTTTAAATATTTTGATACAACAATTGATCGTAAACGATATACGTCTACAAAAGAAGTTTTAGATGCCACTCTTATCCATCAATCCATCACTGGTCTTTATGAAACACGCATTGATTTGAGTCAGCTAGGAGGTGACTGA**

***Streptococcus pyogenes* Cas9 (*Sp*Cas9) amino acid sequence**

**MDKKYSIGLDIGTNSVGWAVITDEYKVPSKKFKVLGNTDRHSIKKNLIGALLFDSGETAEATRLKRTARRRYTRRKNRICYLQEIFSNEMAKVDDSFFHRLEESFLVEEDKKHERHPIFGNIVDEVAYHEKYPTIYHLRKKLVDSTDKADLRLIYLALAHMIKFRGHFLIEGDLNPDNSDVDKLFIQLVQTYNQLFEENPINASGVDAKAILSARLSKSRRLENLIAQLPGEKKNGLFGNLIALSLGLTPNFKSNFDLAEDAKLQLSKDTYDDDLDNLLAQIGDQYADLFLAAKNLSDAILLSDILRVNTEITKAPLSASMIKRYDEHHQDLTLLKALVRQQLPEKYKEIFFDQSKNGYAGYIDGGASQEEFYKFIKPILEKMDGTEELLVKLNREDLLRKQRTFDNGSIPHQIHLGELHAILRRQEDFYPFLKDNREKIEKILTFRIPYYVGPLARGNSRFAWMTRKSEETITPWNFEEVVDKGASAQSFIERMTNFDKNLPNEKVLPKHSLLYEYFTVYNELTKVKYVTEGMRKPAFLSGEQKKAIVDLLFKTNRKVTVKQLKEDYFKKIECFDSVEISGVEDRFNASLGTYHDLLKIIKDKDFLDNEENEDILEDIVLTLTLFEDREMIEERLKTYAHLFDDKVMKQLKRRRYTGWGRLSRKLINGIRDKQSGKTILDFLKSDGFANRNFMQLIHDDSLTFKEDIQKAQVSGQGDSLHEHIANLAGSPAIKKGILQTVKVVDELVKVMGRHKPENIVIEMARENQTTQKGQKNSRERMKRIEEGIKELGSQILKEHPVENTQLQNEKLYLYYLQNGRDMYVDQELDINRLSDYDVDHIVPQSFLKDDSIDNKVLTRSDKNRGKSDNVPSEEVVKKMKNYWRQLLNAKLITQRKFDNLTKAERGGLSELDKAGFIKRQLVETRQITKHVAQILDSRMNTKYDENDKLIREVKVITLKSKLVSDFRKDFQFYKVREINNYHHAHDAYLNAVVGTALIKKYPKLESEFVYGDYKVYDVRKMIAKSEQEIGKATAKYFFYSNIMNFFKTEITLANGEIRKRPLIETNGETGEIVWDKGRDFATVRKVLSMPQVNIVKKTEVQTGGFSKESILPKRNSDKLIARKKDWDPKKYGGFDSPTVAYSVLVVAKVEKGKSKKLKSVKELLGITIMERSSFEKNPIDFLEAKGYKEVKKDLIIKLPKYSLFELENGRKRMLASAGELQKGNELALPSKYVNFLYLASHYEKLKGSPEDNEQKQLFVEQHKHYLDEIIEQISEFSKRVILADANLDKVLSAYNKHRDKPIREQAENIIHLFTLTNLGAPAAFKYFDTTIDRKRYTSTKEVLDATLIHQSITGLYETRIDLSQLGGD***
